# Identification and validation of plasma biomarkers for diagnosis of breast cancer in South Asian women

**DOI:** 10.1038/s41598-021-04176-w

**Published:** 2022-01-07

**Authors:** Thangarajan Rajkumar, Sathyanarayanan Amritha, Veluswami Sridevi, Gopisetty Gopal, Kesavan Sabitha, Sundersingh Shirley, Rajaraman Swaminathan

**Affiliations:** 1 Department of Molecular Oncology, Cancer Institute (WIA), 38, Sardar Patel Road, Chennai, 600036 India; 2 Department of Surgical Oncology, Cancer Institute (WIA), 38, Sardar Patel Road, Chennai, 600036 India; 3 Department of Pathology, Cancer Institute (WIA), 38, Sardar Patel Road, Chennai, 600036 India; 4 Department of Epidemiology and Biostatistics, Cancer Institute (WIA), 38, Sardar Patel Road, Chennai, 600036 India

**Keywords:** Cancer, Molecular biology, Biomarkers, Oncology

## Abstract

Breast cancer is the most common malignancy among women globally. Development of a reliable plasma biomarker panel might serve as a non-invasive and cost-effective means for population-based screening of the disease. Transcriptomic profiling of breast tumour, paired normal and apparently normal tissues, followed by validation of the shortlisted genes using TaqMan^®^ Low density arrays and Quantitative real-time PCR was performed in South Asian women. Fifteen candidate protein markers and 3 candidate epigenetic markers were validated first in primary breast tumours and then in plasma samples of cases [*N* = 202 invasive, 16 DCIS] and controls [*N* = 203 healthy, 37 benign] using antibody array and methylation specific PCR. Diagnostic efficiency of single and combined markers was assessed. Combination of 6 protein markers (Adipsin, Leptin, Syndecan-1, Basic fibroblast growth factor, Interleukin 17B and Dickopff-3) resulted in 65% sensitivity and 80% specificity in detecting breast cancer. Multivariate diagnostic analysis of methylation status of *SOSTDC1*, *DACT2*, *WIF1* showed 100% sensitivity and up to 91% specificity in discriminating BC from benign and controls. Hence, combination of *SOSTDC1*, *DACT2* and *WIF1* was effective in differentiating breast cancer [non-invasive and invasive] from benign diseases of the breast and healthy individuals and could help as a complementary diagnostic tool for breast cancer.

## Introduction

Breast Cancer (BC) is the most common malignancy with an incidence of 24.2% and mortality of 15% among all cancers in women. The mortality rates are higher in transitioning countries including Africa and South Asia, indicating the requirement for effective population based screening programs to detect BC at the earliest^1^. Mammography and ultrasonography are commonly used for detection of breast abnormalities but the sensitivity of these imaging modalities is moderate and results in false negatives (4–34%) particularly in women (under 40 years) with dense breast tissue^2,3^. Additionally, in low-resource setting access to Mammogram can be limited and hence a non-invasive test could help identify those who would need further investigations. Thus, there is an unmet need to develop a strategy that circumvents the shortcomings of the existing methods.

In the past decade, technological advances have facilitated the mining of blood-based BC biomarkers at genomic, transcriptomic, proteomic and metabolomic scale^4–7^. Carcinoembryonic antigen (CEA)^8,9^, human epidermal growth factor receptor-2 (HER2)^10^^,^^11^, the oncogenic protein RS/DJ-1^12^, tissue polypeptide antigen (TPA)^13^ tissue polypeptide specific antigen (TPS)^14^ and CA 15–3^15,16^ have been recommended as biomarkers for metastatic BC, as they were deficient in early BC detection. Amongst the potential multianalyte biomarker panels for early detection of BC^17,18^,a panel of 5 complex autoantigens [B11 (LGALS3), B18 (PHB2), B119 (MUC1), B130 (GK2), and CA15-3], had a high sensitivity of 87% and specificity of 76%^19^.

Parallelly, the analysis of global and gene-specific DNA methylation profiles in circulating cell-free DNA (cfDNA) has led to the discovery of epigenetic markers for BC detection^20^. Liu et al., carried out targeted sequencing of 9223 frequently methylated CpG sites in plasma cfDNA samples of cancer patients. Methylation scores could accurately detect colorectal cancer (96.3%), followed by breast cancer (91.7%), melanoma (81.8%) and NSCLC (61.1%)^21^. The PanSeer assay can simultaneously evaluate a set of differentially methylated genes to detect five cancers (stomach, oesophagus, colorectum, lung or liver). It displayed an overall sensitivity of 95% in detecting the five cancers 0–4 years prior to clinical diagnosis^22^. In BC, the circulating levels of differentially methylated genes such as BRCA1^23^, ATM^24^, CDKN2A^25^, ERα^26^, APC^27^, RASSF1A and RARβ2^28,29^ have also been extensively evaluated in single or combination. The factors limiting the translation of these markers include lack of desired sensitivity and specificity, limited sample size, lack of prospective independent validation, high cost and absence of significant clinical correlation^30^. Thus, the present study aims to develop a combinatorial biomarker assay that serves as a minimally invasive, cost-effective adjunct for BC diagnosis. To achieve this, a transcriptomic profile using microarray was obtained from 41 primary breast tumours (T), 18 paired normal (PN) (adjacent normal tissue to the tumour confirmed to be morphologically normal with no tumour cell present in the sample) and 6 apparent normal (AN) (patients with benign breast disease had breast tissue sampled well away from the lesion) tissues procured from South Asian women. The differentially expressed genes were validated using Quantitative Real-Time Polymerase Chain Reaction (qRT-PCR). Fifteen candidate markers were chosen, and their protein levels were first assessed in tissues [T, PN, AN] and then in plasma of patients with breast cancer (stage 0–4), benign breast abnormalities and in healthy volunteers. The protein marker panel lacked the desired diagnostic power, hence 3 candidate genes which were found to be downregulated in our BC microarray study and confirmed to be methylated in tumours but not in normal breast tissue or in peripheral blood mononuclear cells (PBMC) were chosen for epigenetic marker evaluation. Detection of these methylated genes in cfDNA was performed in the same set of subjects recruited for the protein marker study. The circulating methylation biomarkers achieved 100% sensitivity and up to 90% specificity in discrimination of BC from benign cases and controls.

## Results

### Study population

The clinicopathological details of the samples used for the microarray are given in Supplementary Table S1 and that used in antibody array are furnished in Supplementary Tables S2 and S3 respectively.

### Identification of potential biomarkers using BC gene expression profiling

**Figure 1:**
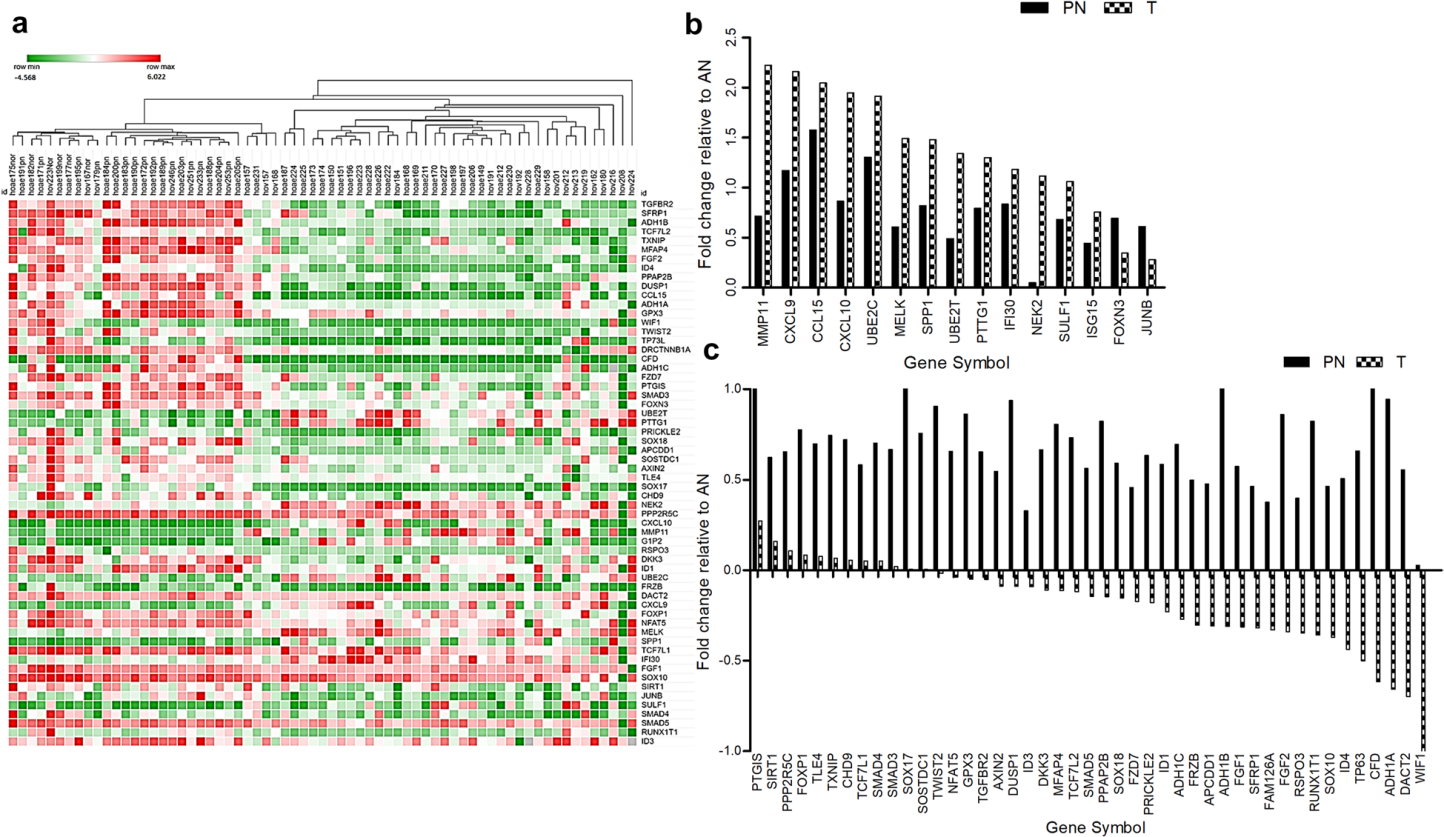
Identification of candidate biomarkers for breast cancer by microarray analyses. (**a**) Unsupervised hierarchical clustering of primary breast tumour, paired normal (PN) and apparently normal (nor) tissues. Each column represents an individual sample and each row a gene. Upregulated genes are indicated in red and downregulated genes in green. (**b**) List of genes upregulated in tumour and paired normal tissues relative to apparently normal (> 2.5-fold differential expression, *P* < *0.0001*) in gene expression profiling. (**c**) List of genes downregulated in tumour and paired normal tissues relative to apparently normal (> 2.5-fold differential expression, *P* < *0.0001*).

Transcriptomic profiling was performed on 41 T,18 PN and 6 AN tissue to obtain a BC gene expression signature (Supplementary Table S1). The raw data analysis using the Class comparison module in the BRB-Array Tools software v 3.7.0 (criteria used were *P*-value = 0.001 and twofold or higher difference) identified 57 genes overexpressed in T compared to either AN or PN, 146 genes with elevated expression in PN compared to AN or T and 173 genes with higher expression in AN compared to PN or T [indicative of downregulated genes in T] (Fig. 1a). Validation of 67 differentially expressed genes (short-listed from microarray analyses) using TaqMan^®^ Low density arrays (TLDA) resulted in 56 genes being identified as differentially expressed, resulting in more than 80% concordance with the microarray data. Fifteen genes were highly overexpressed in T compared to AN and PN; 9 genes were overexpressed in PN compared to AN and T; 32 genes were grossly downregulated in tumours compared to PN or AN samples (Fig. 1b, c). Further, we confirmed the differentially expressed genes from our series using an independent GEO Microarray Dataset [GSE 22820] which included 176 primary breast tumour samples and 10 normal breast tissues from reduction mammoplasties.

Based on the differential expression, 15 markers [CFD, WIF-1, aFGF, bFGF, DKK-3, sFRP-3/FRZB1, LEP, IL-17B, IGF-I, IP-10/CXCL10, LOX-1/OLR1, MIG/CXCL9, MIP-1d/CCL15, OPN/SPP1, SDC1] which were either secreted or likely to be secreted were chosen for further validation.

### Validation of protein biomarkers in BC tissues and plasma

To determine the protein levels of 15 markers, Quantibody array (RayBiotech, USA) based multiplexed sandwich ELISA was first done in tissues (46 T, 23 PN and 6 AN). Among the 15 markers the levels of CFD (*P* = *0.0117*) and LEP (*P* < *0.0001*) were significantly downregulated in T compared to AN and PN. In contrast, 7 out of 15 proteins including, IL17B (*P* = *0.0001*), IP10 (*P* = *0.0135*), LOX-1 (*P* < *0.0001*), MIG (*P* < *0.0001*), MIP-1d (*P* < *0.0001*), SDC1 (*P* < *0.0001*) and OPN (*P* < *0.0001*) were significantly upregulated in tumour tissues compared to normal (AN and PN) (Table 1).

**Table 1:** The expression levels of the 15 candidate biomarkers in breast cancer tissues and normal, estimated by quantibody array.

Protein	Tumour (T)*	Paired normal (PN)*	Apparently normal (AN)*	T versus AN + PN		T versus AN		T versus PN	
	*N* = 46	*N* = 23	*N* = 6	Two tailed Z-value	*P*-value	Two tailed Z-value	*P*-value	Two tailed Z-value	*P*-value
CFD	1046.56 (3.2–30,702)	3532.03 (255–30,402)	14,116.49 (805–23,058)	− 2.52	0.0117	− 1.68	0.093	− 2.2	0.0278
FGF1	12,083.04 (93–93,527)	21,209.35 (4636–55,748)	31,247.21 (0–89,281)	− 1.77	0.0767	− 0.93	0.3524	− 1.65	0.0989
FGF2	20.66 (0–4332)	2.00 (0–3432)	104.74 (0–1205)	1.63	0.1031	− 0.42	0.6745	2.09	0.0366
DKK3	2183.52 (0–19,600)	1098.70 (603–12,889)	5647.57 (0–6399)	1.55	0.1211	− 1.25	0.2113	2.37	0.0178
sFRP3	162.44 (0–15,728)	152.44 (0–6386)	459.12 (0–5619)	− 0.63	0.5287	− 1.53	0.126	− 0.04	0.9681
IGF-I	610.73 (0–2174)	0.00 (0–673)	0.00 (0–78)	1.72	0.0854	1.73	0.0836	1.24	0.215
IL-17B	559.17 (0–3066)	259.81 (0–1297)	31.72 (0–439)	3.9	0.0001	2.11	0.0349	3.62	0.0003
IP-10	58.61 (0–4458)	0.00 (0–21)	0.00 (0–2)	2.47	0.0135	2.38	0.0173	1.83	0.0673
LEP	5.36 (0–315)	6.05 (0–109)	0.00 (0–38)	5.84	< 0.0001	3.14	0.0017	5.43	< 0.0001
LOX-1	15.16 (0–58)	5.10 (0–18)	1.40 (0–6)	5.02	< 0.0001	3.37	0.0008	4.37	< 0.0001
MIG	373.73 (0–9995)	55.11 (0–288)	0.00 (0–92)	5.45	< 0.0001	3.68	0.0002	4.73	< 0.0001
MIP-1d	51.97 (0–326)	9.11 (0–157)	6.39 (0–53)	4.47	< 0.0001	2.42	0.0155	4.14	< 0.0001
OPN	5292.97 (0–142,847)	26.28 (0–1424)	0.00 (0–646)	3.82	0.0001	0.29	0.7718	3.22	0.0013
SDC-1	3746.53 (0–21,503)	158.26 (0–780)	215.38 (0–604)	6.27	< 0.0001	3.44	0.0006	5.8	< 0.0001
WIF1	43.74 (0–386)	0.59 (0–124)	0.00 (0–39)	-0.01	0.992	1.35	0.177	-0.62	0.5353

*The concentration of proteins is expressed in pg/ml and the median values along with range (enclosed in parentheses) is tabulated. Unpaired t-test was used to determine the significance of differential protein levels.

Further, to quantify the levels of these markers in plasma, Quantibody array analysis was carried out on the plasma from patients with invasive (*n* = 202), non-invasive (*n* = 16), benign (*n* = 37) and healthy control subjects (*n* = 203). (Supplementary Fig. S1). Plasma levels of CFD (*P* = *0.002*), LEP (*P* = *0.0629*) and DKK3 (*P* = *0.0116*) were found to be downregulated in patients with Ductal carcinoma in-situ (DCIS) and invasive BC when compared to controls (healthy controls and patients with benign breast conditions). The markers significantly upregulated in BC relative to controls were FGF1 (*P* = *0.001*), FGF2 (*P* = *0.001*), sFRP3 (*P* = *0.007*), IGF-I (*P* = *0.001*), IL-17B (*P* = *0.001*), IP10 (*P* = *0.001*), LOX-1 (*P* = *0.0026*), MIG (*P* = *0.0001*), MIP1d (*P* = *0.0001*), OPN (*P* = *0.0237*), SYN-1 (*P*-*0.0001*), WIF1(*P* = *0.037*) Table 2. Of the 15 markers, the levels of FGF2, sFRP3 and IL17B were highest in the invasive cases relative to DCIS and they gradually declined in benign and control groups. In contrast, LEP levels were lowest in invasive group and the levels gradually increased in DCIS, benign and controls (Supplementary Fig. S2).

**Table 2:** The median (range) concentration of each protein in plasma of cases and controls along with their area under the curve (AUC) value of single markers.

Protein	Controls (*N* = 203)	Benign (*N* = 37)	DCIS (*N* = 16)	Invasive (*N* = 203)	AUC	*P*-value
CFD	15,587.84 (7546–86,020)	13,279.12 (8279–18,671)	13,398.16 (11,712–19,538)	14,956.99 (876–79,219)	.494	*0.0002*
FGF1	453.73 (0–5765)	992.47 (213–11,762)	935.68 (152–2164)	704.56 (0–3669)	.567	< *0.0001*
FGF2	4.38 (0–226)	3.94 (0.7–289)	5.85 (3.7–14.9)	6.44 (0–578)	.628	< *0.0001*
DKK3	52,628.68 (10,914–105,594)	48,443.83 (21,295–68,015)	44,521.15 (16,912–61,505)	48,047.86 (23,045–289,571)	.418	*0.0116*
sFRP3	218.72 (14–3106)	227.95 (48–1780)	232.78 (127–566)	273.27 (31–2994)	.583	*0.0007*
IGF-I	1053.63 (0–70,486)	6675.06 (0–19,616)	6702.13 (1272–10,975)	5143.85 (0–48,697)	.590	< *0.0001*
IL-17B	246.50 (0–1820)	281.29 (0–2572)	644.06 (30–1450)	692.22 (0–102,063)	.655	< *0.0001*
IP-10	793.43 (204–3261)	531.31 (141–3009)	611.60 (225–1174)	872.59 (117–27,803)	.551	< *0.0001*
LEP	16,591.02 (1199–126,621)	15,987.71 (2123–50,503)	15,743.27 (3013–40,763)	15,043.79 (165–100,228)	.455	*0.0629*
LOX-1	31.32 (6.8–588)	46.19 (11–112)	38.50 (14–52)	34.82 (8–388)	.529	*0.0026*
MIG	8.07 (0–1276)	14.59 (1–104)	9.16 (2.8–30)	11.67 (0–1118)	.595	< *0.0001*
MIP-1d	3016.82 (865–30,289)	2105.31 (908–6360)	2959.81 (1518–3714)	3054.95 (896–27,643)	.537	< *0.0001*
OPN	25,970.97 (1429–49,291)	27,841.24 (6272–291,202)	43,285.42 (23,340–26,565)	28,631.63 (2268–285,320)	.519	*0.0237*
Syndecan-1	3433.04 (72–10,157)	3230.24 (1292–15,487)	3016.82 (1798–4169)	4819.05 (174–27,816)	.647	< *0.0001*
WIF1	27.79 (0–6168)	30.87 (0–309)	13.41 (0–131)	43.08 (0–3906)	.584	*0.037*

Kruskal Wallis test was used to calculate significance. Statistical significance (*P* < *0.05*).

### Diagnostic Evaluation of the protein markers in plasma of BC patients

To analyse the diagnostic properties of the protein markers, the plasma samples were split into training (*N* = 371) and test (141) sets (Supplementary Table S4). The individual diagnostic performance of the 15 markers was determined using the receiver operating characteristic (ROC) analysis. The specificity was fixed at 70% and sensitivities were generated. The markers which had an AUC < 0.5 were CFD (35.7 ng/ml, AUC 0.494, 31%), LEP (45.7 ng/ml, AUC 0.455, 29%) and DKK3 (124.6 ng/ml, AUC 0.418, 30%). Markers with AUC > 0.5 were FGF2 (0.0068 ng/ml, AUC 0.628, 47%), IL17B (0.67 ng/ml, AUC 0.655, 52%) and SDC1 (4.565 ng/ml, AUC 0.647, 49%). To improve the sensitivity, multiparameter diagnostic evaluation was performed. The Median + 1 MAD (Median Absolute Deviation) value of each marker across cases and controls set as cut-off to generate sensitivity and specificity. Leave one out biomarker analysis was performed to choose combination with maximum sensitivity. (Supplementary Table S5). Overall, the combination of 6 proteins CFD, LEP, DKK3, IL17B, SDC1, FGF2 showed the best diagnostic potential with sensitivity and specificity of 65% and 79% in training set (AUC 0.661), and 70% and 85% in test set (AUC 0.678). respectively (Table 3). Although the sensitivity of multiple proteins improved the sensitivity of the model, it did not satisfy our objective, hence we evaluated additional markers.

**Table 3:** The diagnostic performance of different multi-marker models in detecting breast cancer.

Biomarker Combination	Training set		Test set	
	Sensitivity (95% CI)	Specificity (95% CI)	Sensitivity (95% CI)	Specificity (95% CI)
CFD + IL17B	40% (32.50–48.68%)	86% (79.94–91.01%)	34% (23.19–45.72%)	91% (81.52–96.64%)
CFD + IL17B + SDC1	67% (58.77–74.32%)	70% (62.92–77.30%)	27% (17.35–38.61%)	98% (91.96–99.96%)
CFD + IL17B + SDC1 + DKK3	56% (47.33–63.70%)	82% (75.22–87.46%)	48% (36.85–60.56%)	92% (83.44–97.53%)
CFD + IL17B + SDC1 + DKK3 + FGF2	71% (62.92–77.96%)	70% (62.92–77.30%)	81% (69.11–89.24%)	71% (64.31–84.90%)
CFD + IL17B + SDC1 + DKK3 + FGF2 + LEP	65% (56.72–72.48%)	79% (71.92–84.85%)	70% (57.73–80.72%)	85% (74.96–92.34%)
CFD + IL17B + SDC1 + DKK3 + FGF2 + LEP + IGF1	74% (66.43–80.94%)	63% (55.43–70.59%)	46% (34.29–57.93%)	97% (89.63–99.64%)
All (15 biomarkers)	94% (88.99–97.24%)	23% (16.74–30.04%)	20% (11.81–31.22%)	100% (94.64–100.00%)

### Identification of Circulating DNA methylation markers

The top downregulated genes [*WIF1, DACT2, ID4, TP63, SOX10*] in tumours in our transcriptomic profile were regulators of Wnt signalling, cell differentiation and morphogenesis and acted as inhibitors of tumour growth in BC^31–34^. Epigenetic silencing of various Wnt signalling antagonists promote abnormal cell proliferation in BC, since they have a tumour suppressive role^35^. Hence, we chose *WIF1* and *DACT2* since they showed the greatest downregulation in T relative to AN (Fig. 1c). Additionally, *SOSTDC1* found to be downregulated in tumours in our gene expression study, had been confirmed to be downregulated in 98.2% (56/57) of breast tumour tissues and cell lines due to promoter hypermethylation in our earlier study, was also included^36^.

### Epigenetic silencing of *WIF1*, *DACT2* and *SOSTDC1* in cell lines and primary breast carcinomas

**Figure 2:**
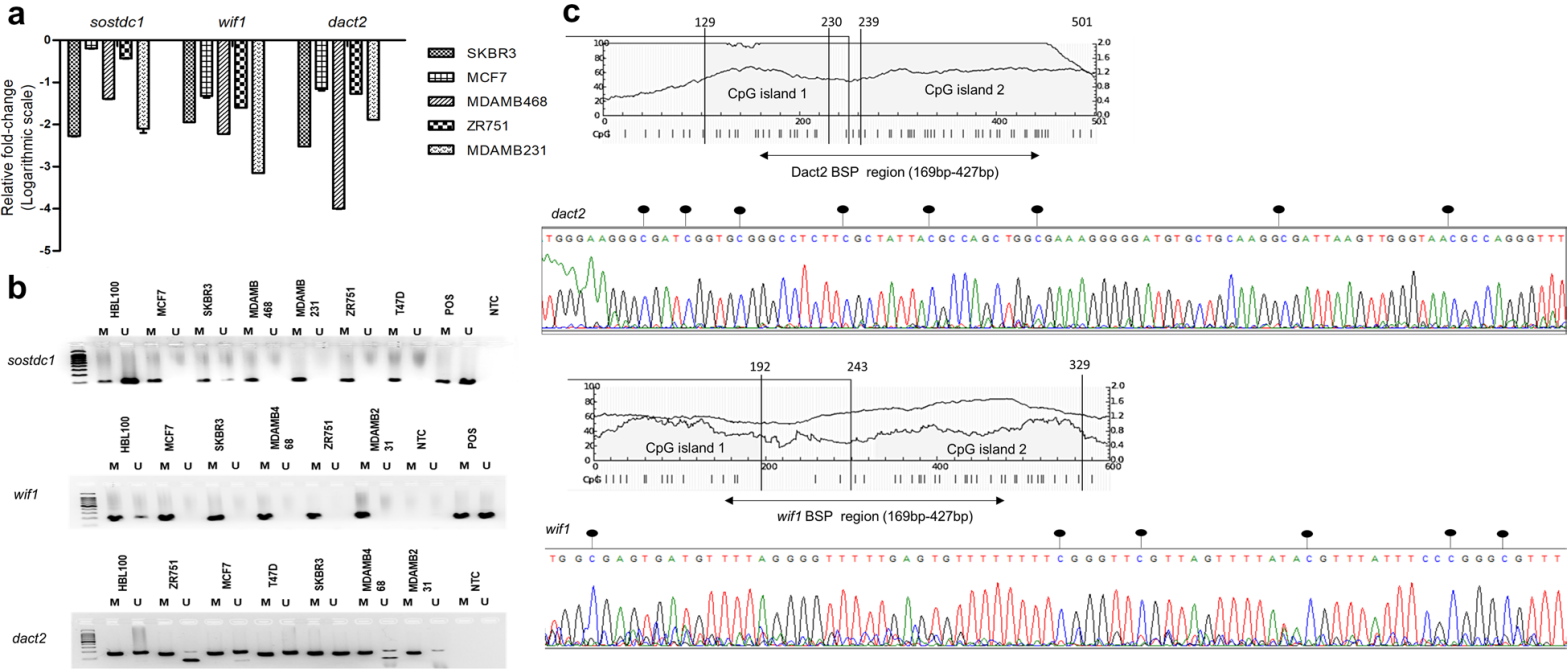
Downregulation of *SOSTDC1*, *DACT2*, *WIF1* in breast cancer cell lines is associated with methylation. (**a**) Expression levels of *SOSTDC1*, *DACT2*, *WIF1* in breast cancer cell lines relative to non-tumorigenic HBL100, analysed by qRT-PCR. The CT values were normalized against the internal control GAPDH. Each sample was run in triplicates and mean ± SD of 2^∆∆CT values were plotted in log scale, (**b**) MSP results of *SOSTDC1*, *DACT2*, *WIF1* in breast cancer cell lines, (**c**) Representative figure of bisulphite gene sequencing of the putative promoter region of *DACT2* and *WIF1* in MDAMB231 cells using gene specific BSP primers. The *DACT2* and *WIF1* amplicons were 257 bp and 493 bp respectively. The black symbols () in chromatogram represent the methylated CpG sites.

To validate the downregulation of *WIF1*, *DACT2* and *SOSTDC1* in various subtypes of BC, qRT-PCR was performed in breast-cancer derived cell lines: ZR751, MCF7, T47D (ER/PR^+^ HER2^-^), SKBR3 (ER/PR^-^ HER^+^), MDAMB231, MDAMB468 (ER/PR^-^ HER2^-^) and a non-tumorigenic breast cell line HBL100^37^. The mRNA levels of all three genes were found to be decreased in BC cell lines with greater downregulation in triple negative MDAMB231, MDAMB468 cells and HER2 overexpressing SKBR3 cells when compared to others (Fig. 2a). We then did Methylation Specific PCR (MSP) analysis and found complete methylation of *WIF1* and *SOSTDC1*, and partial methylation of *DACT2* in all BC cell lines (Fig. 2b).

**Figure 3:**
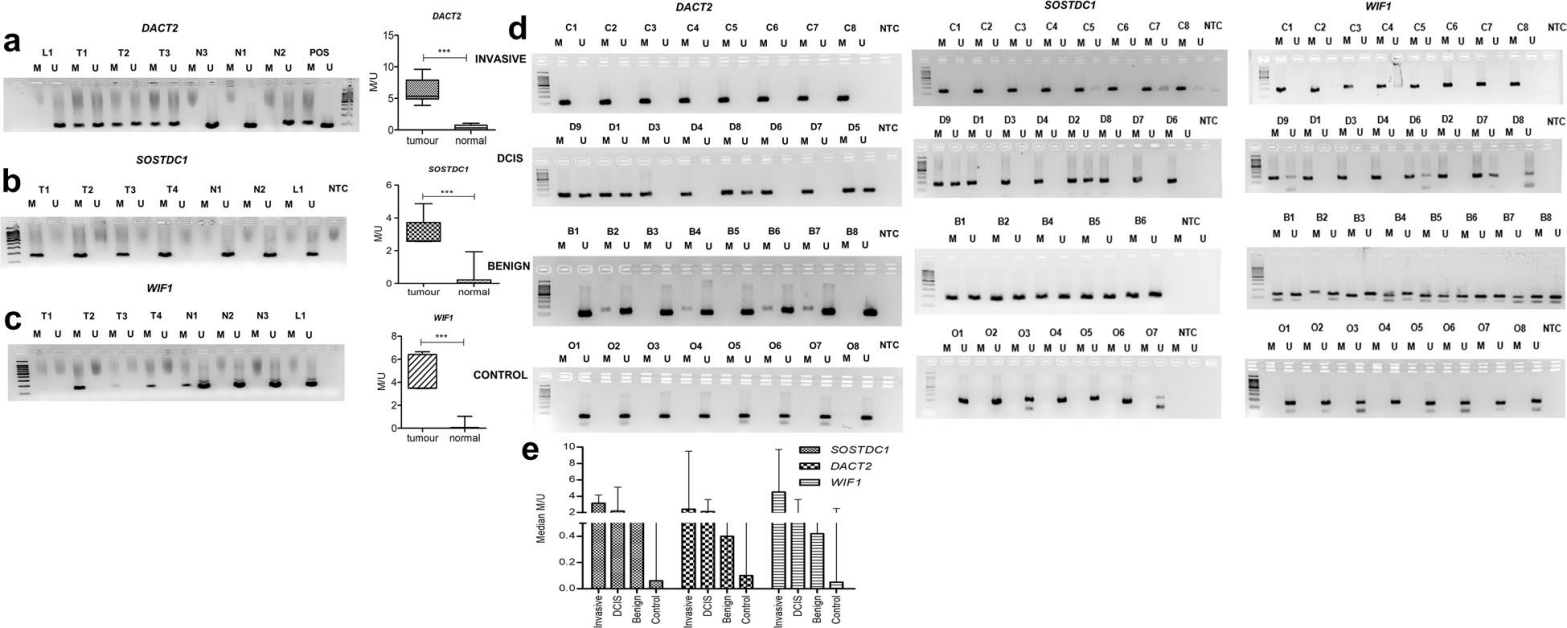
Differential methylation of candidate gene markers in breast cancer. MSP analysis of (**a**) *DACT2*, (**b**) *SOSTDC1*, and (**c**) *WIF1* in primary breast tumour tissues, M/U represents the ratio of methylated allele and unmethylated allele, L-lymphocytes, T-breast tumour tissues, N-paired normal tissues (**d**) MSP analysis of *DACT2, SOSTDC1* and *WIF1* in plasma samples from patients with invasive BC (C = Cancer), non-invasive BC (D = DCIS), benign breast disease (B = benign) and healthy volunteers (O = Controls). The amplicons were resolved in 2% agarose gel. POS- positive control (100% *in-vitro* methylated and unmethylated DNA standards), NTC-no template control, M-methylated allele, U-unmethylated allele. (**e**) Densitometric analysis results of the amplicons obtained from methylation sensitive-PCR. Relative intensity of each band was calculated with 100 bp DNA ladder as reference. M/U represents Ratio of relative intensity of Methylated allele/Unmethylated allele respectively. The median with range intensity was plotted and Kruskal Wallis statistic was used to test the differential methylation intensity. The full-length gels of above images are provided in Supplementary Fig. S4.

Bisulfite Sequencing PCR (BSP) analysis of the putative promoter regions of *WIF1* and *DACT2* uncovered methylated CpG sites, concordant with the MSP analysis (Fig. 2c). The BSP sequencing of *SOSTDC1* was not repeated since, its promoter region was already characterized with the promoter region having a 54 bp CpG island upstream of 5’UTR encompassing 4 CpG sites^38^. Further, the methylation status of these genes was validated in 10 primary breast carcinoma tissues (having > 70% tumour cells) and 10 paired normal tissues. In concordance with the cell lines, *SOSTDC1* and *WIF1* were completely methylated but, *DACT2* was hemi-methylated in tumours. All three markers were unmethylated in the paired normal tissues and lymphocytes confirming that the methylated alleles of *SOSTDC1*, *DACT2* and *WIF1* originate from the breast tumour (Fig. 3a–c).

### *WIF1*, *DACT2* and *SOSTDC1* as potential circulating biomarkers in BC

For the validation of *SOSTDC1*, *DACT2* and *WIF1* in plasma samples, cfDNA was isolated from patients (invasive BC = 202, DCIS = 16, benign = 37) and controls (*n* = 203) recruited for the case control study. Increased concentration of cfDNA was detected in plasma from patients with invasive BC and DCIS when compared to plasma from patients with benign breast diseases and healthy controls (*P* < *0.0001*). cfDNA levels were associated with stage of the disease and were significantly elevated (*P* < *0.0001*) in stage II (Median [M]: 437 ng/ml, Range [R]:236–889), stage III (M: 763 ng/ml, R: 310–2262) and stage IV (M: 1687 ng/ml, R: 421–2447) patients when compared to stage I (M:297 ng/ml, R:279–320) and DCIS (M:221 ng/ml R:178–513) patients. Although the difference in cfDNA levels were not statistically significant between healthy volunteers (M:174 ng/ml, R:66–317), patients with benign breast disease (M:181 ng/ml, R:98–295), DCIS (M:221 ng/ml R:178–513) and early BC (M:297 ng/ml, R:279-320 ng/ml) an increasing trend in the cfDNA concentration was observed (Supplementary Fig. S3). This lack of significance could also be due to the smaller number of DCIS and stage 1 patients included in the study.

MSP detection of *SOSTDC1*, *DACT2* and *WIF1* was carried out with bisulphite modified cfDNA samples isolated from the cases and controls. The amplicons were visualised in 2% agarose gel. (Fig. 3d, Supplementary Fig. 4). *SOSTDC1* was methylated in 95.5% (193/202) of invasive BC patients and 75% (12/16) of DCIS patients, while it was unmethylated in 83.7% (31/37) benign patients and (202/203) in 99.5% of healthy controls. *DACT2* was methylated in 91.08% (184/202) of invasive BC and 75% (12/16) of DCIS patients, and it was unmethylated in 89% (33/37) benign patients and (193/203) in 95.07% of healthy controls. The methylated allele of *WIF1* was detected in 99% (200/202) of invasive BC and 75% of DCIS patients and unmethylated allele was detected in 83.7% (31/37) of benign patients and 98.5% (200/203) of controls. Densitometric analysis was used to obtain the ratio of relative intensity of the methylated and unmethylated alleles (M/U) in each sample. The methylation intensity of *SOSTDC1*, *DACT2* and *WIF1* was significantly elevated (*P* < *0.0001*) in the invasive BC and DCIS, when compared to benign and controls. The benign cases were poorly methylated or unmethylated but a slight increase in methylation intensity was observed in benign lesions with proliferative potential (data not shown). *SOSTDC1* and *DACT2* did show prominent differential methylation in DCIS and invasive BC, but methylated WIF1 was significantly increased (*P* = *0.031*) in DCIS tumours (Fig. 3e, Supplementary Table S6).

### Association of *SOSTDC1*, *DACT2* AND *WIF1* methylation with clinicopathological variables

The association of *SOSTDC1*, *DACT2* AND *WIF1* methylation with the clinicopathological parameters of 218 patients (202 invasive BC, 16 DCIS) in the case control study was studied. Elevated methylation intensity of *SOSTDC1*, *DACT2* and *WIF1* was observed in patients who had advanced tumour stage, positive nodes and local or distant metastasis. In addition, BC patients with grade III had high levels of methylated *SOSTDC1 and DACT2.* However, there was no correlation identified between the methylation patterns of the genes with age, menstrual status and receptor status, with the exception of methylated DACT2 which was high in patients with HER2^-^ BC (Table 4).

**Table 4:** Association of *SOSTDC1*, *DACT2* and *WIF1* methylation with clinicopathological parameters of 202 invasive BC patients and 16 DCIS patients.

Variable	Category	*N*	SOSTDC1 (M/U)	*P*-value	DACT2 (M/U)	*P*-value	WIF1 (M/U)	*P*-value
Age	< 45	79	3.26 (1.76)	0.712	3.5 (4.02)	0.617	4.94 (2.8)	0.639
	≥ 45	139	3.36 (2.0)		3.26 (3.02)		5.16 (3.43)	
Menstrual status	Premenopausal	122	3.18 (1.68)	0.168	3.53 (3.9)	0.287	2.86 (0.25)	0.655
	Post-menopausal	86	3.56 (2.89)		2.93 (1.9)		3.07 (0.33)	
Tumor Stage	0-II	92	2.69 (2.04)	< 0.0001*	2.7 (2.5)	0.0212*	2.68 (1.3)	< 0.0001*
	III-IV	126	3.85 (1.63)		3.81 (3.9)		6.8 (3.1)	
Tumor grade	I-II	72	2.87 (1.96)	0.0103*	3.31 (0.50)	0.76	4.22 (0.38)	0.0014*
	III	136	3.57 (1.77)		3.46 (0.27)		5.69 (0.26)	
Nodal status	Negative	24	2.42 (2.92)	0.017*	1.99 (0.79)	0.027*	2.43 (0.13)	< 0.0001*
	Positive	184	3.46 (1.78)		3.06 (3.8)		5.39 (2.9)	
Metastasis	Negative	173	2.98 (0.12)	< 0.0001*	2.957 (0.21)	< 0.0001*	4.29 (0.21)	< 0.0001*
	Positive	35	4.987 (0.33)		5.819 (0.96)			
ER receptor	Negative	69	3.39 (1.63)		2.95 (2.74)		5.002 (2.707)	
	Positive	127	3.4 (2.13)	0.95	3.38 (3.11)	0.337	5.17 (3.38)	0.709
PR receptor	Negative	109	3.47 (1.76)	0.505	3.507 (3.51)	0.286	4.89 (3.49)	0.338
	Positive	87	3.29 (2.165)		3.05 (2.63)		5.33 (2.96)	
HER2 receptor	Negative	52	2.58 (0.94)	0.523	4.61 (4.2)	0.015*	6.002 (3.68)	0.268
	Positive	42	3.43 (1.12)		2.97 (2.34)		5.018 (3.21)	
	2 +	108	3.43 (1.9)		2.67 (0.309)		5.003 (1.51)	

t-test and one-way analysis of variance (ANOVA) was used to test the differential methylation among various clinic-pathological parameters, *P-value < 0.05 denotes statistical significance. The Mean M/U value along with standard deviation in parentheses is tabulated for each parameter. M-methylated allele, U-unmethylated allele.

### Diagnostic evaluation of *SOSTDC1*, *DACT2* AND *WIF1* as a multi-marker panel for BC

To analyse the diagnostic performance of the predicted diagnostic model, invasive BC, DCIS patients were clubbed as cases and the benign and healthy controls were combined as controls. The samples were randomly split into training and test sets. Univariate ROC analysis of the markers in the training set (151 cases, 166 controls) revealed an AUC of 0.993 (95% CI 0.9866–0.9997) for *SOSTDC1*, AUC of 0.994 (95% CI 0.9898–0.9987) for *DACT2*, AUC of 0.9966 (95% CI 0.9930–1.000) for *WIF1*. Multivariate ROC analysis of the 3 gene panel in the training set displayed an AUC of 1.000 (95% CI 1.000–1.000). The markers were validated in an independent set of samples (67 cases, 73 controls) for testing the accuracy of the markers. In the test set, univariate ROC analysis showed an AUC of 0.994 (95% CI 0.9873–1.002) for *SOSTDC1*, AUC of 0.983 (95% CI 0.9687–0.9976) for *DACT2* and AUC of 0.995 (95% CI 0.9902–1.001) for *WIF1*. The combined AUC of 3 genes was 1.000 (95% CI 1.000–1.000) (Supplementary Fig. S5). The overall performance of the markers in single and in combination were concordant in the training and test sets. Hence, individually the markers indicated excellent discriminatory power and when combined, the 3 markers were able to detect BC with 100% efficiency.

To confirm the accuracy of the markers, an arbitrary cut-off of ‘1’ was assigned and leave one out analysis was performed using the training set. In cases, if M/U (ratio of methylated allele and unmethylated allele) value is greater than 1, a score of ‘1’ was assigned and if M/U value is lesser than 1, a score of ‘0’ was assigned. In controls, if M/U value is greater than 1, a score of ‘-1’ was assigned and if M/U value is lesser than 1, a score of ‘0’ was assigned. The diagnostic parameters were calculated using MEDCALC^®^ tool. In the training set, individually all three markers showed > 90% sensitivity and specificity, with an accuracy of 94% for *SOSTDC1* (91.17- 96.60), 94% for DACT2 (90.42–96.10) and 97% for WIF1 (93.88–98.26). The combination of DACT2 with SOSTDC1 displayed a sensitivity and specificity of 100% (97.59–100.00) and 91% (85.53–94.85); DACT2 with WIF1 showed a sensitivity of 100% (97.59–100) and specificity of 91% (86.25–95.31). The 3 gene signature showed a sensitivity and specificity of 100% (97.59–100.00) and 88% (82.70–92.97). In the test set, *SOSTDC1* and *WIF1* could discriminate BC cases with approximately 95% sensitivity and specificity, but *DACT2* exhibited only 85% sensitivity. All the markers individually displayed > 90% specificity in the test set, in concordance with the training set data. In the multivariate analysis, a combination of *SOSTDC1*, *DACT2* and *WIF1* yielded 100% (94.64–100.00) sensitivity, 89% (79.80–95.22) specificity and accuracy 94.33% (89.13–97.52) (Table 5).

**Table 5:** Discriminant analysis of single gene and multi-gene biomarkers in breast cancer.

Biomarker combination	Training set		Test set	
	Sensitivity (95% CI)	Specificity (95% CI)	Sensitivity (95% CI)	Specificity (95% CI)
DACT2	92% (86.53–95.83%)	95% (90.73–97.90%)	85% (74.26–92.60%)	92% (83.18–96.97%)
SOSTDC1	94% (88.99–97.24%)	94% (89.96–97.49%)	94% (85.41–98.35%)	97% (90.58–99.67%)
WIF1	98% (94.30–99.59%)	95% (90.73–97.90%)	95% (87.47–99.07%)	99% (92.70–99.97%)
DACT2 + SOSTDC1	100% (97.59–100.00%)	91% (85.53–94.85%)	100% (94.64–100.00)	89% (79.80–95.22%)
WIF1 + SOSTDC1	99% (96.37–99.98%)	92% (86.98–95.76%)	97% (89.92–99.65%)	100% (95.01–100.00%)
DACT2 + WIF1	100% (97.59–100.00%)	91% (86.25–95.31%)	97% (89.63–99.64%)	90% (81.48–96.11%)
All (3 markers)	100% (97.59–100.00%)	88% (82.70–92.97%)	100% (94.64–100.00%)	89% (79.80–95.22%)

Thus, combining multiple markers improved the overall sensitivity of the predicted diagnostic model.

## Discussion

In the last few years several attempts have been made to develop a non-invasive assay for the early diagnosis of BC. In recent times, the use of multiple markers for BC detection is gaining interest because, the combination of functionally important genes and proteins involved in breast tumorigenesis, have exhibited better diagnostic accuracy than single candidate markers^30^. A 3-protein diagnostic model named Mastocheck was able to predict BC with a sensitivity, specificity of 71.58%, 85.25% and AUC 0.8323^39,40^. A tumour suppressor methylation panel (*APC, BIN1, BMP6, BRCA1, CST6, ESR-b, GSTP1, P16, P21 and TIMP3*) showed 91.7% sensitivity in distinguishing BC patients from normal individuals^41^. A 6 epimarker panel (*SFN, P16, hMLH1, HOXD13, PCDHGB7*, RASSF1a) discriminated BC and benign cases from controls with 78% sensitivity^42^ and combinatorial methylation panel comprising *GSTP1, RARβ2, RASSF1* and *APC* detected early-stage tumours with a sensitivity of 33%. In addition, SNiPER gene panel showed a sensitivity of 63% for DCIS and 51% early invasive tumour detection at 80% specificity^43^. Apart from these, attempts at targeted sequencing of ubiquitously methylated CpG sites have made it possible to detect and classify multiple cancer types from plasma cfDNA^21,22^.

In the present study, we have aimed at developing a minimally invasive, multi-marker BC diagnostic model with improved sensitivity and specificity in South Asian women. Transcriptomic profiling followed by qRT PCR validation identified 56 candidate markers for biomarker evaluation. Fifteen of the 56 genes were then analysed at the protein level using a multiplexed sandwich ELISA platform (Quantibody array), first in tissues and later in plasma. Eight markers (FGF2, IL17B, IP10, MIG, MIP1d, LOX1, OPN and SDC1) found to be upregulated in primary tumours were present in higher concentration in plasma of breast cancer patients. Similarly, 3 markers (CFD, LEP and DKK3) were downregulated both in tumour lysates and plasma from patients with cancer. Hence, the expression of 11 out 15 markers showed similar trend in tumour tissues and in plasma. IGF-1 induces Ras-Raf-MAPK and PI3K-AKT signalling components to promote tumour progression and elevated levels of serum IGF-1 was associated with poor prognosis^44–46^. Consistent with the existing literature, IGF-1 mRNA and protein levels were found to be higher in BC in our study population. The mitogenic growth factors FGF1 and FGF2, known to possess potent angiogenic properties^47,48^, were increased in patients with malignant breast tumours and decreased in patients with benign breast lesions indicating their possible role in malignant cell transformation. IL17B, is produced by induction of memory T lymphocytes and plays an important role in inflammatory responses by binding to the membrane receptor IL17RB. IL17B-IL17RB interaction triggers the activation of NF-kB signalling cascade leading to production of anti-apoptotic Bcl-2^49^. IL17B levels were significantly higher in our DCIS and invasive mammary carcinoma patients and diminished in benign and control groups. The protein SDC1 was also significantly increased in our BC cases, on par with previous findings which have demonstrated that high serum levels of SDC1 were associated with aggressive phenotype, poor prognosis and decreased response to chemotherapy^50–52^. In majority of studies, increased levels of CFD and LEP have been linked to obesity associated BC progression via enhanced TGFβ signalling and MMP modulation^53–55^. In contrast, our results indicate decreased levels of the adipokines, CFD and LEP in BC tissues as well as plasma when compared to controls but it is similar to a transcriptome profiling study in Arabian women that has reported the downregulation of leptin and other downstream leptin metabolism genes in BC^56^.

The dysregulation of WNT signalling cascade in tumorigenesis has been well documented and WNT antagonists sFRP3, DKK3 and WIF1 are frequently downregulated in BC contributing to constitutive activation of oncogenic growth factors^35^. Our data depicts decreased plasma levels of DKK3 in cases which correlate with the previous gene expression data but the median protein levels of WIF1 were higher in BC tissues and plasma, contrary to our microarray results. Inter-individual variability of WIF1 was observed to be high in our data, and WIF1 levels were below the detection limit in 30–40% of the plasma samples, hence the median and normal range was difficult to establish making it less reliable. Univariate ROC analysis of the markers showed limited sensitivity and specificity, so markers which had AUC above or below 0.5 were chosen for multivariate analysis. Combination of 6 proteins including CFD, IL17B, SDC1, DKK3, FGF2, LEP showed a sensitivity of 65%, 70% and specificity of 79% and 85% in training and test sets respectively. The multi-protein panel possessed higher discriminant performance than single markers but did not achieve the desired sensitivity and specificity.

Next, we assessed the methylation status of *SOSTDC1*, *DACT2* and *WIF1* in cfDNA samples isolated from reserved aliquots of plasma from cases and controls recruited. All three genes are known negative modulators of the canonical Wnt signalling cascade. The deficiency of *SOSTDC1* correlated with greater tumour size and treatment with recombinant *SOSTDC1* effectively blocked WNT signalling components which contribute to cell proliferation, indicating its antagonistic role against Wnt pathway^57^. In addition, our previous study reported that *SOSTDC1* is downregulated in 98.1% of breast tumour tissues and it coincides with DNA methylation of CpG sites in promoter region of *SOSTDC1*^36^. Epigenetic inactivation of *WIF1* contributes to constitutive activation of WNT signalling pathway in breast tumorigenesis^58^. The differential methylation of *WIF1* was also able to predict the clinical efficacy of neoadjuvant chemotherapy (docetaxel, pirarubicin and cyclophosphamide) in sera of locally advanced BC patients^59^. *DACT2* acts as a tumour suppressor by inhibiting canonical WNT signalling and is frequently silenced by promoter hypermethylation in BC. Overexpression of *DACT2* inhibited the expression of β-catenin target genes associated with tumour growth and metastasis^60,61^. Although these 3 genes were known to be frequently methylated in breast tumour tissues, their methylation status in circulating DNA remained unknown. Therefore, *SOSTDC1*, *DACT2* and *WIF1* seemed candidate markers for the development of a potential methylation-based diagnostic model.

Our data showed reduced mRNA levels and methylation of the 3 genes in breast cancer cell lines and tumour tissues consistent with previous studies which have reported that promoter methylation mediated silencing of *WIF1* and *DACT2* was observed in 63.3% (95/150)^62^ and 73% (107/147)^33^ of primary BC tissues.

BSP analysis was then carried out to confirm the methylation status in a methylation-independent manner. Consequently, the putative promoter methylation of the 3 markers was analysed in cfDNA isolated from plasma of patients diagnosed with either non-invasive or invasive BC, benign breast diseases and healthy individuals. The markers of interest were found to be methylated in more than 90% of invasive and 75% of pre-invasive BC cases. They were either unmethylated or weakly methylated in more than 80% of the benign cases and 95% of healthy controls. The clinicopathological correlation revealed that rise in methylation intensity of the said genes were significantly associated with advanced tumour stage, high grade, nodal status, and metastases. These results signify the putative tumour suppressive role of the markers and possible role in progression and severity of BC.

The receptor status of breast tumours did not show any influence on the methylation levels except for *DACT2* which displayed significant variation among tumours which were HER2 positive and negative. The HER2 receptor status of 50% of the patients had a score of 2 + and FISH confirmation was not done for all due to financial issues (Supplementary Table S7). Diagnostic evaluation of the single markers displayed a high sensitivity range of 92%-98% and specificity of 95% in the training set. In the test set, the sensitivity and specificity of the single markers ranged from 85 to 95% and 92–99% respectively. Combination of 2 or 3 biomarkers generated 100% sensitivity and 89–91% specificity in both the training and test datasets. Hence, the predicted diagnostic model was robust and reliable since it could discriminate BC (non-invasive and invasive) from plasma obtained from patients with benign breast disease and healthy controls with a better sensitivity than the previously proposed models.

Despite, the promising results of various biomarker panels reported previously, the current study has some strengths. Although multiple markers show better diagnostic potential than single markers, it is imperative to establish an assay comprising of minimal number of markers with high diagnostic ability. In addition, the assay platform should also be cost-effective and easily adaptable in a clinical setting. In this study we were able to achieve 97–100% sensitivity and 89–91% specificity by combining 2 or 3 genes. We have used MSP, a PCR based semi-quantitative platform which is simple and cost-effective compared to sequencing and mass spectrometric approaches. Pre-analytical parameters such as use of plasma instead of serum [plasma is preferred for cfDNA assays since serum cfDNA has increased genomic DNA levels^63,64^ and a three-step centrifugation step to avoid lymphocyte contamination was incorporated. Unlike many of the existing studies the specificity of our panel was also tested in benign breast abnormalities and DCIS and validated in an independent sample set. The differential methylation of the said epigenetic markers, between the benign, DCIS and invasive groups suggest that these markers shall be able to differentiate benign lesions from malignant. Although the epigenetic silencing of *WIF1*, *DACT2* and *SOSTDC1* in BC has been reported before, we are the first team to evaluate the combined diagnostic efficiency of these genes in circulating cfDNA from BC patients.

The shortcoming of our study is the limited number of DCIS and early-stage BC samples which can contribute to the lack of statistical significance in the differential methylation intensity between controls, benign and early-stage tumours (DCIS and stage I). Hence, validation of these markers in a larger number of DCIS and early-stage breast tumour patients is necessary before it is considered as a complementary tool for BC diagnosis.

## Materials and methods

### Patients and samples

The study was approved by the Institute Ethical committee, Cancer Institute (WIA). The study population primarily consisted of Indian women. The patient samples were collected after informed consent and all methods were performed in accordance with relevant guidelines and regulations set by the committee. Our Institute’s Tumour bank provided 6 apparent normal (AN) samples; 41 breast tumours (T) with at least 70% tumour cells in the sample provided as confirmed by frozen section and 18 paired normal (PN) samples that were included in the microarray study and for qRT-PCR validation of the microarray data. For the validation of the markers identified, the protein levels were initially estimated in BC tissue lysates comprising of 6 AN, 23 PN and 46 T samples (Supplementary Table S1). The frequency of women opting for reduction mammoplasty or for prophylactic mastectomy is very rare in India, hence it is difficult to procure normal breast tissue samples. Our work hence used samples well away from the benign lesions and confirmed to be morphologically normal, from women with benign breast disease.

Further, an age-matched case–control study (age distribution with 5-year intervals) was performed with 202 BC patients and 203 healthy controls. The inclusion and exclusion criteria are provided in (Supplementary Table S6). Additionally, 16 patients with ductal carcinoma *in-situ* (DCIS) and 37 patients with benign breast disease were included in the study. The samples were randomly split into training set and test set for validation (Supplementary Fig. S1).

### Sample preparation

The tissue lysates were prepared according to Rajkumar et al.^65^. The concentration of proteins was estimated in 100ul of lysate (1 mg/ml). Post clinical examination, 10 ml blood sample was collected in ethylene diamine tetra acetic acid (EDTA) coated tubes from each individual. The cells and plasma were separated from whole blood by centrifugation at 2500 rpm for 20 min at 37℃ and stored in aliquots in LoBind tubes [Eppendorf, India] at − 80 °C. The plasma samples were thawed and centrifuged at 13,000 rpm and 100 µl (1:1) of diluted cell-free plasma was added to each well of the custom designed Quantibody array slide (Raybiotech, Inc. USA).

### Cell lines

The cell lines HBL100, MCF7, MDAMB231, MDAMB468, ZR751, SKBR3 and T47D were used. All the cell lines were purchased from National Centre for Cell Science (Pune, India).

### Nucleic acid extraction

Genomic DNA was isolated from cell lines, tissues samples (breast tumour, paired normal and apparent normal) by QIAamp^®^ DNA Mini Kit (Qiagen, Hilden) according to manufacturer’s instructions. Cell free DNA was isolated from 1 ml of plasma using QIAamp Circulating Nucleic Acid Kit (Qiagen, Hilden). DNA and RNA quantification were done using NanoDrop ND1000 (NanoDrop Technologies, USA) spectrophotometer. Cell free DNA was quantitated using Qubit™ dsDNA HS Assay Kit (Invitrogen, USA).

The RNA was extracted from the tissue samples using the RNeasy RNA extraction kit (Qiagen, Hilden; Cat no: 74106) as per the manufacturer’s instructions. The quality of the RNA used for microarray analysis was checked using the Bioanalyzer and samples with RNA Integrity Number (RIN) of 7 or more were included in the study. RNA was quantitated using NanoDrop ND1000 spectrophotometer (NanoDrop Technologies, USA).

### Microarray based gene expression profiling

The microarray experiments were done as described previously^65^. Briefly, 1 µg of total RNA from the tumour/PN/AN sample and universal RNA (Stratagene; Cat no: 740000–41) were reverse transcribed using Array script at 42 °C for 2 h to obtain cDNA using the Amino Allyl MessageAmp II aRNA amplification kit (Ambion, Austin, Tx; Cat no: AM1797). The cDNA was amplified, labelled, hybridized and slides scanned as described earlier. All the raw data files have been submitted to GEO (accession number GSE139038).

### Microarray data analysis

The microarray data analysis was done as described previously using BRB-ArrayTools software v 3.7.0 (http://linus.nci.nih.gov/BRB-ArrayTools.html)65. The differentially expressed genes among the 3 classes (tumour/PN/AN) were analysed using the Class comparison module of the BRB-Array Tools software. Univariate F-test was used and the genes were considered statistically significant if their *P*-value was < 0.001. In addition, a two-fold difference in gene expression was required between the different classes [T vs. PN vs. AN].

### Quantitative real time PCR

Validation of the gene expression was done using the TLDA quantitative real time PCR (Applied Biosystems, Foster City, CA). Triplicate cDNA template samples were amplified and analysed on the ABI Prism 7900HT sequence detection system (Applied Biosystems, Foster City, CA). The protocol for validation was adapted from literature^65^. The raw data from the Prism 7900HT sequence detection system was imported into Microsoft Excel for statistical analysis of the data. Among the endogenous reference genes included on the array (18S ribosomal gene; UBC; GAPDH), UBC and GAPDH were chosen after visualizing the global Ct value distribution, for data normalization. The TLDA assays were run at Lab India Instruments Pvt Ltd laboratories at Gurgaon, New Delhi.

The AN tissue samples were used as calibrators and the relative quantitation values were calculated for all the genes and the samples. Geometric mean was calculated for each of the 67 genes (excluding the 3 endogenous controls). The relative quantitation values for all the samples and genes were imported into BRB Array Tools and Class Comparison analysis was done comparing the different clinicopathological parameters with the gene expression values. Fifteen genes which were secreted or potentially secreted were short-listed, based on our data as well as corroborated with GEO Microarray Dataset (176 primary breast tumors and 10 normal breast samples [from reduction mammoplasties] [GSE22820]). An additional requirement was that the protein could be assayed in the multiplexed sandwich-ELISA platform.

### Custom designed protein antibody array (quantibody array)

Quantibody array [multiplexed sandwich ELISA on a glass slide] was used [Ray Biotech, Inc, USA] to study the protein expression in tissue lysates and subsequently in plasma. The assay was done as per the manufacturer’s instructions with modifications^65^.

### Quantibody array data normalization

The data was analysed using the H15S90 Genxbio Q-Analyzer v8 16.4, an array specific, Microsoft Excel based program, supplied with the custom arrays. 2 positive controls for signal normalization, a negative control for background subtraction and an internal control to minimize inter-slide variation were used. A user defined reference array was used, to which the signals of other arrays were normalized. Median + Median absolute deviation was calculated for each protein and the cut-offs which yielded a sensitivity and specificity of more than 75% was chosen.

### cDNA conversion and semiquantitative RT-PCR

1 µg of RNA was used to synthesize first strand cDNA (QuantiTect Reverse Transcription Kit, Qiagen, Hilden). The reaction mixture was diluted 50% (v/v) with water and 2 µl of cDNA was used for 25 µl PCR reaction (Eurogentec Takyon™ Low ROX SYBR^®^ 2X Mastermix Blue). RT-PCR was amplified for 35 cycles and GAPDH was used as an internal control for normalization. The products were visualized in 2% agarose gel.

### Bisulfite conversion, methylation specific PCR (MSP) and bisulfite sequencing PCR (BSP) analysis

Bisulfite conversion was performed using the EZ DNA Methylation-Gold Kit (Zymo Research, USA), according to manufacturer’s instructions. The amount of input DNA was adjusted to be uniform for each sample. For MSP and BSP analysis of Dapper 2 homolog (*DACT2)*, genomic sequence 1000 bp upstream of transcription start site (TSS) and 200 bp downstream of TSS containing the putative promoter region was retrieved from Database of transcriptional start sites (http://dbtss.hgc.jp/). MethPrimer 2.0 (http://www.urogene.org/methprimer2/) was used for CpG island prediction and generation of primer sets. For Sclerostin domain containing 1 (*SOSTDC1*)^36^ and Wnt Inhibitory Factor 1 (*WIF1*)^62^*,* pre-designed primer sets (spanning promoter CpG islands) from previous studies were used (Supplementary Table S8). HotStar Taq^®^ Master Mix (Qiagen, Hilden, Germany) was used for MSP and the conditions were set according to manufacturer’s instructions. The amplicons were resolved in 2% agarose gel.

BSP products of *WIF1* and *DACT2* were amplified from bisulfite modified MDAMB231 genomic DNA using HotStar Taq^®^ with gene-specific primers (Supplementary Table S8) and cloned using TOPO^®^ TA Cloning^®^ Kit for Sequencing (Invitrogen, Massachusetts USA). The clones were screened using PCR and restriction enzyme digestion. The clones were sequenced using a Big Dye Terminator Cycle Sequencing kit (Applied Biosystems, Foster city, CA, USA) as per the manufacturer’s instructions on ABI 310 genetic analyser. Densitometric analysis was used to quantify (Image Lab 6.0.1.) the intensity of the DNA bands relative to 100 bp DNA ladder (Promega, Madison USA). Ratio of methylated and unmethylated band intensity was calculated for each sample in cases and controls. Median + Median absolute deviation was calculated for each gene and used as cut-offs.

### Equipment and settings

The Quantibody array slides were scanned at 5 μm resolution, ratio (635/532) and a PMT of 580 using Molecular Devices GenePix Pro 4100 A scanner. At these scanner settings the signals from the highest standard concentration did not reach saturation.

The MSP amplicon resolved agarose gels were docked using Biorad ChemiDoc MP system. Image Lab version 4.1 software (Bio Rad CA, USA, https://www.bio-rad.com/en-in/product/image-lab-software)^66^ was used for gel imaging with acquisition settings such as single channel, SyBR Safe mode and gray scale image colour. The image exposure and image area were set as default. For relative quantification of band intensity, Promega 100 kb DNA ladder was used as reference standard.

### Statistical analysis

Statistical tests were done using XLSTAT version 2018.1, (Microsoft, WA, USA) https://www.xlstat.com^67^ and Prism v5.01 (GraphPad software, CA,USA, https://www.graphpad.com). Kruskal Wallis Test was employed to test significance of differential protein levels and methylation intensity. Unpaired t-test and One-way analysis of variance was used to assess association of relative methylation levels of BC patients with clinicopathological parameters. Statistical significance was defined as *P* < *0.05*. The ROC curve analysis and binomial logistic regression was performed using IBM SPSS for Windowsversion 28 (IBM Corp, NY, USA https://www.ibm.com/in-en). The multivariate ROC was generated using Predicted Probabilities module in Binomial Logistic Regression.

### Ethics declarations

The study was approved by the Institute’s Ethical committee (IEC), Cancer Institute (WIA) by their letter dated 16–06–2007 (for use of tissue samples from Tumour Bank) and letter dated 18–08–2014 (for case – control study). Patient samples were collected after informed consent and the study was performed in compliance to the National Ethical Guidelines for Biomedical Health Research involving Human Participants and conditions of Indian Council of Medical Research.

## Supplementary Information

Supplementary Information.

## Data Availability

The dataset generated in this study is publicly available in Gene Expression Omnibus, [https://www.ncbi.nlm.nih.gov/geo/query/acc.cgi?acc=GSE139038]. The dataset used in the current study is publicly available in Gene Expression Omnibus, [https://www.ncbi.nlm.nih.gov/geo/query/acc.cgi?acc=GSE22820].

## References

[1] Bray F (2018). Global cancer statistics 2018: GLOBOCAN estimates of incidence and mortality worldwide for 36 cancers in 185 countries. CA. Cancer J. Clin..

[2] Schopper D, de Wolf C (2009). How effective are breast cancer screening programmes by mammography? Review of the current evidence. Eur. J. Cancer.

[3] Wang L (2017). Early diagnosis of breast cancer. Sens. (Switz.).

[4] Li J (2020). Non-invasive biomarkers for early detection of breast cancer. Cancers (Basel)..

[5] Hanash SM, Pitteri SJ, Faca VM (2008). Mining the plasma proteome for cancer biomarkers. Nature.

[6] Kim J (2021). Identification of MicroRNAs as diagnostic biomarkers for breast cancer based on the cancer genome atlas. Diagnostics.

[7] Yang L (2020). Application of metabolomics in the diagnosis of breast cancer: a systematic review. J. Cancer.

[8] Pathak KA (1996). Carcinoembryonic antigen : an invaluable marker for advanced breast cancer. J. Postgrad. Med..

[9] Shao Y, Sun X, He Y, Liu C, Liu H (2015). Elevated levels of serum tumor markers CEA and CA15-3 are prognostic parameters for different molecular subtypes of breast cancer. PLoS ONE.

[10] Ross JS, Fletcher JA (1998). The HER-2/neu oncogene in breast cancer: Prognostic factor, predictive factor, and target for therapy. Stem Cells.

[11] Pedersen AC, Sørensen PD, Jacobsen EH, Madsen JS, Brandslund I (2013). Sensitivity of CA 15–3, CEA and serum HER2 in the early detection of recurrence of breast cancer. Clin. Chem. Lab. Med..

[12] Oda M (2012). High levels of DJ-1 protein in nipple fluid of patients with breast cancer. Cancer Sci..

[13] Liwowska I, Kopczyński Z, Grodecka-Gazdecka S (2006). Diagnostic value of measuring serum CA 15–3, TPA, and TPS in women with breast cancer. Postepy. Hig. Med. Dosw..

[14] D’Alessandro R (2001). Serum tissue polypeptide specific antigen (TPS): a complementary tumor marker to CA 15–3 in the management of breast cancer. Breast Cancer Res. Treat..

[15] Hashim ZM (2014). The significance of CA15-3 in breast cancer patients and its relationship to HER-2 receptor status. Int. J. Immunopathol. Pharmacol..

[16] Di Gioia D (2015). Serum HER2 in combination with CA 15–3 as a parameter for prognosis in patients with early breast cancer. Clin. Chim. Acta.

[17] Winden AWJO, Rodenburg W, Pennings JLA (2012). A bead-based multiplexed immunoassay to evaluate breast cancer biomarkers for early detection in pre-diagnostic serum. Int. J. Mol. Sci..

[18] Kazarian A (2017). Testing breast cancer serum biomarkers for early detection and prognosis in pre-diagnosis samples. Nat. Publ. Gr..

[19] Zuo X (2016). Identification of a panel of complex autoantigens (LGALS3, PHB2, MUC1, and GK2) in combination with CA15-3 for the diagnosis of early-stage breast cancer. Tumor Biol..

[20] Tang Q, Cheng J, Cao X, Surowy H, Burwinkel B (2016). Blood-based DNA methylation as biomarker for breast cancer: a systematic review. Clin. Epigenetics.

[21] Liu L (2018). Targeted methylation sequencing of plasma cell-free DNA for cancer detection and classification. Ann. Oncol..

[22] Chen X (2020). Non-invasive early detection of cancer four years before conventional diagnosis using a blood test. Nat. Commun..

[23] Bosviel R (2012). BRCA1 promoter methylation in peripheral blood DNA was identified in sporadic breast cancer and controls. Cancer Epidemiol..

[24] Flanagan JM (2009). Gene-body hypermethylation of ATM in peripheral blood DNA of bilateral breast cancer patients. Hum. Mol. Genet..

[25] Delmonico L (2015). CDKN2A (p14ARF/p16INK4a) and ATM promoter methylation in patients with impalpable breast lesions. Hum. Pathol..

[26] Hagrass HA, Pasha HF, Ali AM (2014). Estrogen receptor alpha (ERα) promoter methylation status in tumor and serum DNA in Egyptian breast cancer patients. Gene.

[27] Salta S (2018). A DNA methylation-based test for breast cancer detection in circulating cell-free DNA. J. Clin. Med..

[28] Kloten V (2013). Promoter hypermethylation of the tumor-suppressor genes ITIH5, DKK3, and RASSF1A as novel biomarkers for blood-based breast cancer screening. Breast Cancer Res..

[29] Skvortsova TE (2006). Cell-free and cell-bound circulating DNA in breast tumours: DNA quantification and analysis of tumour-related gene methylation. Br. J. Cancer.

[30] Loke SY, Lee ASG (2018). The future of blood-based biomarkers for the early detection of breast cancer. Eur. J. Cancer.

[31] Tong X (2014). SOX10, a novel HMG-box-containing tumor suppressor, inhibits growth and metastasis of digestive cancers by suppressing the Wnt/β-catenin pathway. Oncotarget.

[32] Nasif D (2018). Epigenetic regulation of ID4 in breast cancer: tumor suppressor or oncogene?. Clin. Epigenetics.

[33] Xiang T (2016). DACT2 silencing by promoter CpG methylation disrupts its regulation of epithelial-to-mesenchymal transition and cytoskeleton reorganization in breast cancer cells. Oncotarget.

[34] Wissman C (2003). WIFI, a component of the Wnt pathway, is down-regulated in prostate, breast, lung, and bladder cancer. J. Pathol..

[35] Suzuki H (2008). Frequent epigenetic inactivation of Wnt antagonist genes in breast cancer. Br. J. Cancer.

[36] Rawat A, Gopisetty G, Thangarajan R (2014). E4BP4 is a repressor of epigenetically regulated SOSTDC1 expression in breast cancer cells. Cell. Oncol..

[37] Dai X, Cheng H, Bai Z, Li J (2017). Breast cancer cell line classification and its relevance with breast tumor subtyping. J. Cancer.

[38] Gopal G, Raja UM, Shirley S, Rajalekshmi KR, Rajkumar T (2013). SOSTDC1 down-regulation of expression involves CpG methylation and is a potential prognostic marker in gastric cancer. Cancer Genet..

[39] Kim Y (2019). Mastocheck: notable plasma protein biomarker for diagnosis of breast cancer in the real clinical practice by using multiple reaction monitoring-based mass spectrometry. J. Clin. Oncol..

[40] Kim Y (2019). Efficacy of mastocheck for screening of early breast cancer: comparison with screening mammography. J. Breast Dis..

[41] Radpour R (2011). Hypermethylation of tumor suppressor genes involved in critical regulatory pathways for developing a blood- based test in breast cancer. PLoS ONE.

[42] Shan M (2016). Detection of aberrant methylation of a six-gene panel in serum DNA for diagnosis of breast cancer. Oncotarget.

[43] Mijnes J (2019). Sniper: a novel hypermethylation biomarker panel for liquid biopsy based early breast cancer detection. Oncotarget.

[44] Peyrat JP (1993). Plasma insulin-like growth factor-1 (IGF-1) concentrations in human breast cancer. Eur. J. Cancer.

[45] Fuchuang Z, Suling L (2020). Mechanistic insights of adipocyte metabolism in regulating breast cancer progression. Pharmacol. Res..

[46] Derek L, Roberts CT (2003). The insulin-like growth factor system and cancer. Cancer Lett..

[47] Slattery ML (2013). Associations with growth factor genes (FGF1, FGF2, PDGFB, FGFR2, NRG2, EGF, ERBB2) with breast cancer risk and survival: the Breast Cancer Health Disparities Study. Breast Cancer Res. Treat..

[48] Faridi A (2002). Long-term follow-up and prognostic significance of angiogenic basic fibroblast growth factor (bFGF) expression in patients with breast cancer. Pathol. Res. Pract..

[49] Alinejad V, Dolati S, Motallebnezhad M, Yousefi M (2017). The role of IL17B-IL17RB signaling pathway in breast cancer. Biomed. Pharmacother..

[50] Malek-Hosseini Z, Jelodar S, Talei A, Ghaderi A, Doroudchi M (2017). Elevated Syndecan-1 levels in the sera of patients with breast cancer correlate with tumor size. Breast Cancer.

[51] Barbareschi M (2003). High syndecan-1 expression in breast carcinoma is related to an aggressive phenotype and to poorer prognosis. Cancer.

[52] Götte M (2006). Predictive value of syndecan-1 expression for the response to neoadjuvant chemotherapy of primary breast cancer. Anticancer Res..

[53] Goto H (2019). Adipose-derived stem cells enhance human breast cancer growth and cancer stem cell-like properties through adipsin. Oncogene.

[54] Pan H (2018). Association between serum leptin levels and breast cancer risk. Med. (U. S.).

[55] Cirillo D, Rachiglio AM, La Montagna R, Giordano A, Normanno N (2008). Leptin signaling in breast cancer: An overview. J. Cell. Biochem..

[56] Karim S (2016). Low expression of leptin and its association with breast cancer: a transcriptomic study. Oncol. Rep..

[57] Clausen KA (2011). SOSTDC1 differentially modulates Smad and beta-catenin activation and is down-regulated in breast cancer. Breast Cancer Res. Treat..

[58] Ai L (2006). Inactivation of Wnt inhibitory factor-1 (WIF1) expression by epigenetic silencing is a common event in breast cancer. Carcinogenesis.

[59] Han Z, Xu C, Han HUI, Wang C, Lin S (2017). Value of the level of methylation of RASSF1A and WIF-1 in tissue and serum in neoadjuvant chemotherapeutic assessment for advanced breast cancer. Oncol. Lett..

[60] Guo L (2018). Methylation of DACT2 contributes to the progression of breast cancer through activating WNT signaling pathway. Oncol. Lett..

[61] Li J (2017). Methylation of DACT2 promotes breast cancer development by activating Wnt signaling. Sci. Rep..

[62] Veeck J (2009). Prognostic relevance of Wnt-inhibitory factor-1 (WIF1) and Dickkopf-3 (DKK3) promoter methylation in human breast cancer. BMC Cancer.

[63] Trigg RM, Martinson LJ, Parpart-Li S, Shaw JA (2018). Factors that influence quality and yield of circulating-free DNA: a systematic review of the methodology literature. Heliyon.

[64] Schmidt B, Fleischhacker M (2018). Is liquid biopsy ready for the litmus test and what has been achieved so far to deal with pre-analytical issues?. Transl. Cancer Res..

[65] Rajkumar T, Vijayalakshmi N, Gopal G (2010). Identification and validation of genes involved in gastric tumorigenesis. Cancer Cell Int..

[66] Colella AD (2012). Comparison of Stain-Free gels with traditional immunoblot loading control methodology. Anal. Biochem..

[67] Costantini A (2018). Predictive role of plasmatic biomarkers in advanced non-small cell lung cancer treated by nivolumab. Oncoimmunology.

[68] Sathyanaryanan, A. *et al.* Identification of Potential Epigenetic Biomarkers for Breast Cancer. in *IACR 2019 38th Annual Convention of Indian Association for Cancer Research* 140 (2019).

[69] Rajkumar, T., Sathyanaryanan, A., Gopisetty, G., Veluswami, S. & Kesavan, S. Blood based biomarkers for early diagnosis and follow up of breast cancer. (2020).

