# Supplementary material for: Identification and validation of plasma biomarkers for diagnosis of breast cancer in South Asian women
Source: Sci Rep. 2022 Jan 7;12:100. doi: 10.1038/s41598-021-04176-w (PMC8742108; doi:10.1038/s41598-021-04176-w)
Supplement: Supplementary file 1 — Supplementary Information. [file 41598_2021_4176_MOESM1_ESM.docx]

**IDENTIFICATION AND VALIDATION OF PLASMA BIOMARKERS FOR DIAGNOSIS OF BREAST CANCER**

THANGARAJAN RAJKUMAR^1,^ *, SATHYANARAYANAN AMRITHA^1^, SRIDEVI VELUSWAMI^2^, GOPISETTY GOPAL^1^, KESAVAN SABITHA^1^, SUNDERSINGH SHIRLEY^3^, RAJARAMAN SWAMINATHAN^4^.

1 – Dept. of Molecular Oncology

2 – Dept. of Surgical Oncology

3 – Dept. of Pathology

4 – Dept. of Epidemiology and Biostatistics

* - Corresponding Author

Dr.T.Rajkumar,

Prof. and Head,

Dept. of Molecular Oncology,

Cancer Institute (WIA),

38, Sardar Patel Road, Chennai – 600036, India

Fax: 91-44-24912085; Tel : 91-44-22350340

Email: [drtrajkumar@gmail.com](about:blank)

**Supplementary Table S1**

The clinicopathological characteristics of the patient samples used for breast cancer microarray analysis

| **Parameters** | **Sub-category** | **Tumor** | **Paired normal (PN)** | **Apparently normal**  **(AN)** |
| --- | --- | --- | --- | --- |
| Age | 30-39 | 2 | 2 | 3 |
|  | 40-49 | 14 | 3 | 1 |
|  | 50-59 | 13 | 5 | 1 |
|  | 60-69 | 9 | 6 | 1 |
|  | 70-79 | 3 | 2 | - |
| Stage | I | 2 |  |  |
|  | II | 25 |  |  |
|  | III | 12 |  |  |
|  | IV | 2 |  |  |
| Histopathology | IDC | 38 |  |  |
|  | ILC | 3 |  |  |
|  | Morphologically  normal |  | 18 | 6 |
| ER | Positive | 26 |  |  |
|  | Negative | 15 |  |  |
| PR | Positive | 22 |  |  |
|  | Negative | 19 |  |  |
| HER2 | Positive | 18 |  |  |
|  | Negative | 22 |  |  |
|  | Unknown | 1 |  |  |
| Molecular Subtype | Luminal | 26 |  |  |
|  | HER2 enriched | 9 |  |  |
|  | TNBC | 5 |  |  |
|  | Unknown | 1 |  |  |

**Supplementary Table S2**

The clinicopathological characteristics of invasive and non-invasive breast cancer patients recruited for quantibody array analysis

| **Characteristic** | **Invasive Breast Cancer** | **Training set** | **Test set** | **DCIS** | **Training set** | **Test set** |
| --- | --- | --- | --- | --- | --- | --- |
|  | 20-24 |  |  | 20-24 |  |  |
| Age | 25-29 | 2 | 2 | 25-29 |  |  |
|  | 30-34 | 9 | 4 | 30-34 |  |  |
|  | 35-39 | 17 | 9 | 35-39 |  | 1 |
|  | 40-44 | 20 | 11 | 40-44 | 3 |  |
|  | 45-49 | 33 | 17 | 45-49 | 4 | 2 |
|  | 50-54 | 22 | 8 | 50-54 | 2 | 1 |
|  | 55-59 | 23 | 7 | 55-59 | 2 | 1 |
|  | 60-64 | 9 | 4 | 60-64 |  |  |
|  | 64-69 | 5 |  | 64-69 |  |  |
| Stage | I | 2 | 2 |  |  |  |
|  | II | 53 | 19 |  |  |  |
|  | III | 65 | 33 |  |  |  |
|  | IV | 20 | 8 |  |  |  |
|  |  |  |  |  |  |  |
| Menstrual status | Premenopausal | 66 | 36 | Premenopausal | 5 | 2 |
|  | Perimenopausal | 11 | 3 | Perimenopausal | 1 |  |
|  | Postmenopausal | 63 | 23 | Postmenopausal | 5 | 3 |
|  |  |  |  |  |  |  |
| Grade | I | 2 | 6 | Low | 2 | 2 |
|  | II | 40 | 27 | Intermediate | 1 | 1 |
|  | III | 98 | 30 | High | 8 | 2 |
|  |  |  |  |  |  |  |
| Histology | Invasive Ductal Carcinoma | 119 | 49 | comedo | 1 |  |
|  | Invasive Lobular Carcinoma | 13 | 7 | cribiform |  |  |
|  | Mixed | 4 | 3 | comedo+cribiform | 1 | 1 |
|  | Poorly diffrentiated Carcinoma | 4 | 4 | not known | 9 | 4 |
|  |  |  |  |  |  |  |
| Nodal status | N0 | 6 | 4 | N0 | 8 | 4 |
|  | N1 | 80 | 35 | N1 | 3 | 1 |
|  | N2 | 42 | 15 |  |  |  |
|  | N3 | 12 | 8 |  |  |  |
|  |  |  |  |  |  |  |
| Metastasis | Metastasis positive | 30 | 14 | Metastasis positive |  |  |
|  | Metastasis negative | 110 | 49 | Metastasis negative | 11 | 5 |
|  |  |  |  |  |  |  |
| Tumor Size | T1 | 9 | 5 | Tis | 9 | 3 |
|  | T2 | 57 | 20 | T1mic | 2 | 2 |
|  | T3 | 24 | 12 |  |  |  |
|  | T4 | 50 | 26 |  |  |  |
|  |  |  |  |  |  |  |
| Receptor status | ER positive | 82 | 45 | ER positive | 2 |  |
|  | ER negative | 58 | 11 | ER negative | 4 | 4 |
|  | PR positive | 66 | 21 | PR positive | 2 | 2 |
|  | PR negative | 79 | 30 | PR negative | 4 | 2 |
|  | HER2 positive | 29 | 13 | HER2 positive | 3 | 2 |
|  | HER2 2+ | 74 | 34 | HER2 2+ | 1 | 1 |
|  | HER2 negative | 37 | 15 | HER2 negative | 2 | 1 |
|  | Ki67 low (≤15%) | 8 | 6 | Ki67 low (≤15%) | 1 | 2 |
|  | Ki67 intermediate (16-25%) | 8 | 5 | Ki67 intermediate (16-25%) | 1 | 1 |
|  | Ki67 high (>25%) | 122 | 52 | Ki67 high (>25%) | 4 | 1 |
|  |  |  |  | Unknown | 5 | 1 |
| Molecular subtype | Luminal | 82 | 45 | Luminal | 2 | 1 |
|  | HER2 enriched | 17 | 4 | HER2 enriched | 3 | 2 |
|  | TNBC | 10 | 9 | TNBC | 1 | 1 |
|  | Unclassified (ER/PR- HER2 2+) | 30 | 5 | Unclassified (ER/PR- HER2 2+) | - | - |

**Supplementary Table S3**

The characteristics of benign breast disease and healthy controls recruited for quantibody array analysis

| Characteristic | Benign | Training set | Test set | Controls | Training set | Test set |
| --- | --- | --- | --- | --- | --- | --- |
|  | 20-24 |  | 1 | 20-24 | 2 | 2 |
| Age | 25-29 | 2 | 1 | 25-29 | 9 | 4 |
|  | 30-34 | 4 | 3 | 30-34 | 17 | 9 |
|  | 35-39 | 8 | 1 | 35-39 | 20 | 11 |
|  | 40-44 | 5 | 2 | 40-44 | 33 | 17 |
|  | 45-49 | 4 | 1 | 45-49 | 22 | 8 |
|  | 50-54 | 2 | 2 | 50-54 | 23 | 7 |
|  | 55-59 |  |  | 55-59 | 9 | 4 |
|  | 60-64 | 1 | 1 | 60-64 | 5 |  |
|  | 64-69 |  |  | 64-69 |  |  |
| Menstrual status | Premenopausal | 21 | 6 |  | 66 | 37 |
|  | Perimenopausal | 1 | 1 |  | 7 | 2 |
|  | Postmenopausal | 4 | 4 |  | 67 | 24 |
|  |  |  |  |  |  |  |
| Histology | Atypical Hyperplasia | 3 |  |  |  |  |
|  | Proliferative lesions without atypia | 3 |  |  |  |  |
|  | Fibroadenoma | 5 | 8 |  |  |  |
|  | Fibroepithelial lesion | 3 | 1 |  |  |  |
|  | Fibrocycstic changes | 9 | 1 |  |  |  |
|  | Benign phyllodes tumor | 2 | 1 |  |  |  |
|  | Hemangioma | 1 |  |  |  |  |

**Supplementary Table S4**

The median concentration of 15 proteins in training sets and test set samples obtained by Quantibody array analysis

| **Protein** | **Training Set** | | **Test Set** | |
| --- | --- | --- | --- | --- |
|  | **Cases (pg/ml)**  **(140 invasive + 11 dcis)** | **Controls (pg/ml)**  **(140 normals +**  **26 benign)** | **Cases (pg/ml)**  **(62 invasive +**  **5 dcis)** | **Controls (pg/ml)**  **(63 normals +**  **11 benign)** |
| CFD | 14210.76 | 15772.75 | 14905.60 | 16725.06 |
| FGF1 | 706.96 | 449.53 | 828.78 | 695.80 |
| FGF2 | 6.71 | 3.83798288 | 5.80 | 3.64 |
| DKK3 | 46185.93 | 48403.134 | 48627.35 | 59680.92 |
| sFRP3 | 291.25 | 217.3 | 236.48 | 217.24 |
| IGF-I | 5761.89 | 1550.575 | 5412.38 | 1710.12 |
| IL-17B | 837.99 | 393.775 | 689.20 | 231.83 |
| IP-10 | 877.97 | 696.139768 | 1007.64 | 813.71 |
| LEP | 13013.52 | 15512.0253 | 15231.31 | 17960.77 |
| LOX-1 | 32.97 | 31.87 | 40.66 | 37.79 |
| MIG | 11.91 | 8.775 | 9.97 | 7.91 |
| MIP-1d | 2611.63 | 2521.6886 | 3459.62 | 3310.14 |
| OPN | 25324.66 | 22810.79 | 37784.31 | 32060.59 |
| Syndecan-1 | 4081.12 | 3121.25387 | 5606.62 | 3815.37 |
| WIF1 | 49.12 | 32.33 | 25.16 | 20.55 |

**Supplementary Table S5**

**The scoring criteria for diagnostic evaluation of protein markers**

| **Markers** | **Training Set Cut-off (Median +1MAD)** | **Test Set Cut-off (Median +1MAD)** | **Scoring in Controls (Healthy and Benign)** | **Scoring in Cases**  **(Invasive and DCIS)** |
| --- | --- | --- | --- | --- |
| CFD (Decreased in cases) | 16438.87 | 16487.11 | >cut-off 1, <cut-off 0 | >cut-off -1, <cut-off 0 |
| FGF1 (Increased in cases) | 806.14 | 1064.75 | >cut-off -1, <cut-off 0 | >cut-off 1,  <cut-off 0 |
| FGF2 (Increased in cases) | 6.07 | 5.89 | >cut-off -1, <cut-off 0 | >cut-off 1,  <cut-off 0 |
| DKK3 (Decreased in cases) | 56285.23 | 56329.75 | >cut-off 1, <cut-off 0 | >cut-off -1, <cut-off 0 |
| sFRP3 (Increased in cases) | 318.00 | 304.62 | >cut-off -1, <cut-off 0 | >cut-off 1,  <cut-off 0 |
| IGF-I (Increased in cases) | 3101.15 | 3420.23 | >cut-off -1, <cut-off 0 | >cut-off 1,  <cut-off 0 |
| IL-17B (Increased in cases) | 749.98 | 423.93 | >cut-off -1, <cut-off 0 | >cut-off 1,  <cut-off 0 |
| IP-10 (Increased in cases) | 984.97 | 1043.17 | >cut-off -1, <cut-off 0 | >cut-off 1,  <cut-off 0 |
| LEP (Decreased in cases) | 18593.47 | 18777.05 | >cut-off 1, <cut-off 0 | >cut-off -1, <cut-off 0 |
| LOX-1 (Increased in cases) | 44.53 | 49.85 | >cut-off -1, <cut-off 0 | >cut-off 1,  <cut-off 0 |
| MIG (Increased in cases) | 14.10 | 13.04 | >cut-off -1, <cut-off 0 | >cut-off 1,  <cut-off 0 |
| MIP-1d (Increased in cases) | 3431.24 | 4108.17 | >cut-off -1, <cut-off 0 | >cut-off 1,  <cut-off 0 |
| OPN (Increased in cases) | 35933.61 | 47101.74 | >cut-off -1, <cut-off 0 | >cut-off 1,  <cut-off 0 |
| Syndecan-1 (Increased in cases) | 4296.68 | 4889.19 | >cut-off -1, <cut-off 0 | >cut-off 1,  <cut-off 0 |
| WIF1 (Increased in cases) | 54.85 | 41.09 | >cut-off -1, <cut-off 0 | >cut-off 1,  <cut-off 0 |

**Supplementary Table S6**

The median relative intensity of methylated markers in cases and controls. *Kruskal-Wallis test of significance was used to assess the differential methylation levels among the groups

| **Gene** | **Controls**  **(N=203)** | **Benign**  **(N=37)** | **DCIS**  **(N=16)** | **Invasive**  **(N=203)** | **P-value*** |
| --- | --- | --- | --- | --- | --- |
| Sostdc1 | 0.06 (0-1.09) | 0.4 (0.05-1.38) | 2.18 (0.2-5.1) | 3.14 (0.6-4.16) | <0.0001 |
| Dact2 | 0.1 (0-1.82) | 0.4 (0.06-1.12) | 2.12 (0.7-3.6) | 2.41 (0.63-9.5) | <0.0001 |
| Wif1 | 0.05 (0-2.5) | 0.42 (0.04-1.62) | 1.82 (0.7-3.6) | 4.52 (0.8-9.7) | <0.0001 |

**Supplementary Table S7**

The enrolment criteria set for recruitment of patients and controls for the case-control study

|  | **INCLUSION CRITERIA** | **EXCLUSION CRITERIA** |
| --- | --- | --- |
| **CASES** | Histopathologically confirmed breast carcinoma.  Should provide informed consent  Age between 20 and 70 years  No prior history of treatment for breast cancer. | Unwilling to provide informed consent.  Severe co-morbid conditions including uncontrolled diabetes, hypertension, Systemic autoimmune disorders including SLE, Scleroderma, Rheumatoid arthritis etc.  HIV, Hepatitis B or C positivity  Individuals on steroids or NSAIDs on long term basis. |
| **CONTROLS** | No prior history of benign breast disease.  No clinical evidence of benign breast disease  Should provide informed consent  No history of mastectomy, hysterectomy or oophorectomy  No first or second-degree relatives with breast or ovarian or prostate cancers. | Failure to satisfy all the inclusion criteria  Severe co-morbid conditions including uncontrolled diabetes, hypertension, Systemic autoimmune disorders including SLE, Scleroderma, Rheumatoid arthritis etc.  HIV, Hepatitis B or C positivity  Individuals on steroids or NSAIDs on long term basis. |

**Supplementary Table S8**

List of the primers used in the study

| **GENE** | **PRIMER SEQUENCE (5’ – 3’)** | **AMPLICON SIZE (BP)** | **REFERENCE** |
| --- | --- | --- | --- |
| *wif1*  methylated | F: CGTTTTATTGGGCGTATCGT  R: ACTAACGCGAACGAAATACGA | 145 | (1) |
| *wif1* unmethylated | F: GGGTGTTTTATTGGGTGTATTGT  R: AAAAAAACTAACACAAACAAAATACAAAC | 154 | (1) |
| *dact2* methylated | F: TTGGTTATAGATTTTAGTTTATTTTGGC  R: TATATCGCGAATCCTCCTACG | 80 | (2) |
| *dact2* unmethylated | F: GTTATAGATTTTAGTTTATTTTGGTGA  R: AACTATATCACAAATCCTCCTACACC | 80 | (2) |
| *sostdc1* methylated | F: TTTTTTAAATGAATAGCGATGTATTT TC  R: TTCATCACTTATCTATAAACCG ACG | 136 | (3) |
| *sostdc1* unmethylated | F: TTTTTTAAATGAATAGTGATGTATTTTTGT  R: ATCACTTATCTATA AACCAACACA | 135 | (3) |
| *wif1* RT-PCR | F: AGATCCAACCGTCAATGTCC  R:  ACCCCATCCTGTTTTCCAAG | 143 | (4) |
| *Dact2* RT-PCR | F: CCCCTGTTTGTCCTGACTAAG  R: CCTGTCGATATAAGCTCTGGC | 149 | (4) |
| *Sostdc1* RT-PCR | F: ATGACAAAACCCGTACCCAG  R: CTGTGATTTTGTAGGTGCGTG | 162 | (4) |
| *Sostdc1* BSP | F: GAAAGTTAGTTT TTTTAAATGAATAG  R: AAACTTCATCACTT ATCTATAAACC | 493 | (3) |
| *Dact2* BSP | F: GGGGGAGGTYGYGGTGATTT  R: ACCTACRACRATCCCAACCC | 254 | (5) |
| *Wif1* BSP | F: TTATTATTAGTATTTAGTTAAGTTT  R: ACCTAAATACCAAAA AACCTA | 493 | (6) |

**Supplementary File S1:** **Abstract submitted for guest lecture in the 40th Annual Conference of Indian Association of Cancer Research**

BIOMARKERS FOR EARLY DIAGNOSIS OF BREAST CANCER

Thangarajan Rajkumar^1^, *, Sathyanarayanan Amritha^1^, Sridevi Veluswami^2^, Gopisetty Gopal^1^, Kesavan Sabitha^1^, Sundersingh Shirley^3^, Rajaraman Swaminathan^4^

1 – Dept. of Molecular Oncology

2 – Dept. of Surgical Oncology

3 – Dept. of Pathology

4 – Dept. of Epidemiology and Biostatistics

Breast cancer is the most common cancer among Indian women. While the outlook for early breast cancers is good, locally advanced and metastatic breast cancers have lower survival rates. The data from the Institute shows that the case distribution is as follows [2013] – Stage 1 -10; stage 2A – 49, 2B – 160; 3A – 142; 3B – 111; 3C – 25; IV – 65; Stage unknown – 5 [total 567 cases]. The 5-year DFS for breast cancer in stage I, II, III, IV and Staging not possible [SNP – Operated outside] is 82, 81, 60, 18 and 77, respectively. Therefore, there is an urgent need for simple non-invasive tests for early diagnosis.

We share our work on identifying potential markers for early diagnosis of breast cancer.

**Supplementary Fig S1: Schematic representation of subject recruitment for case-control study**

**
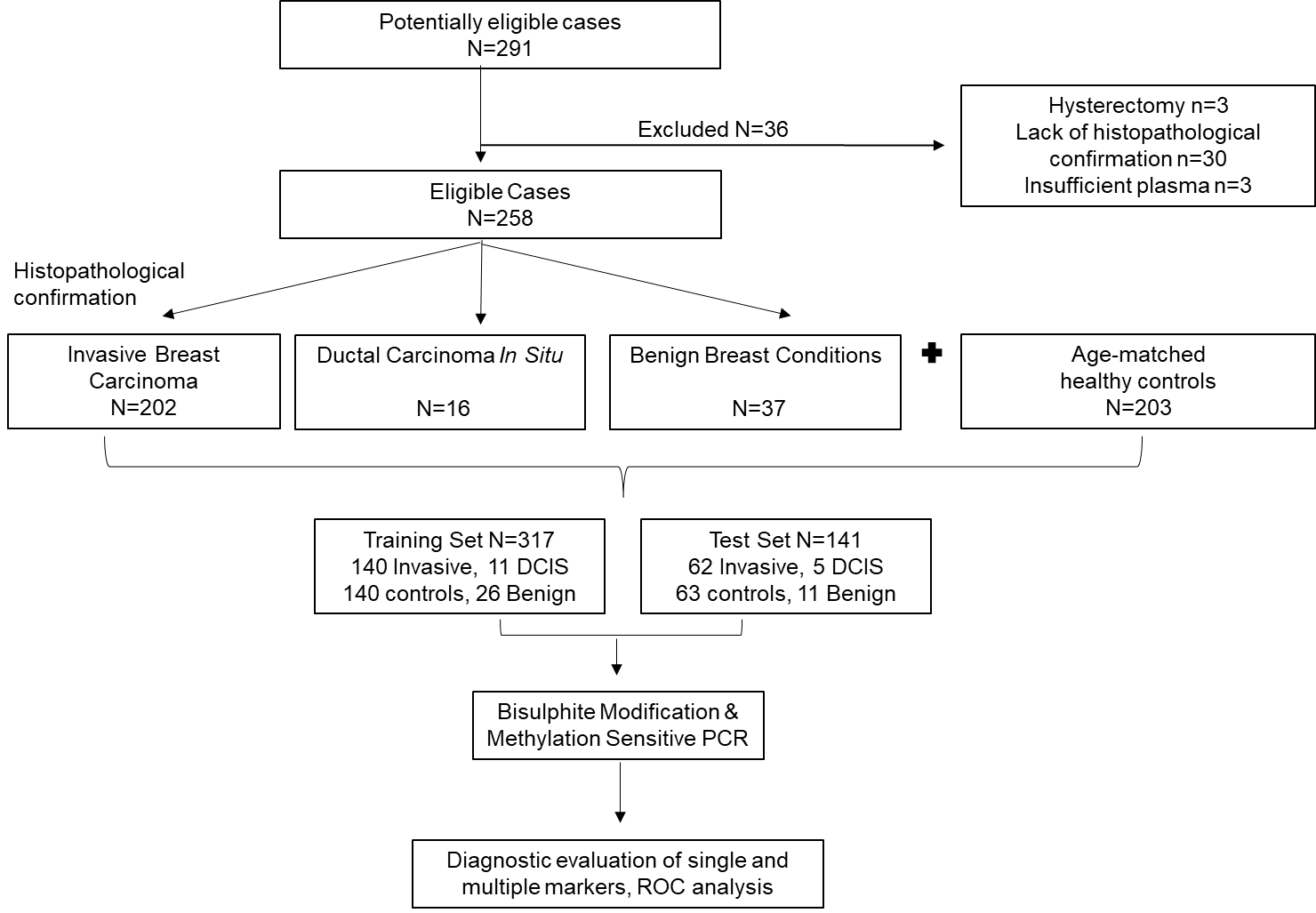
**

**Supplementary Fig S2** **The levels of markers showing an increasing trend in cases vs controls. The median concentration of proteins in each group is plotted to obtain a trendline**

**
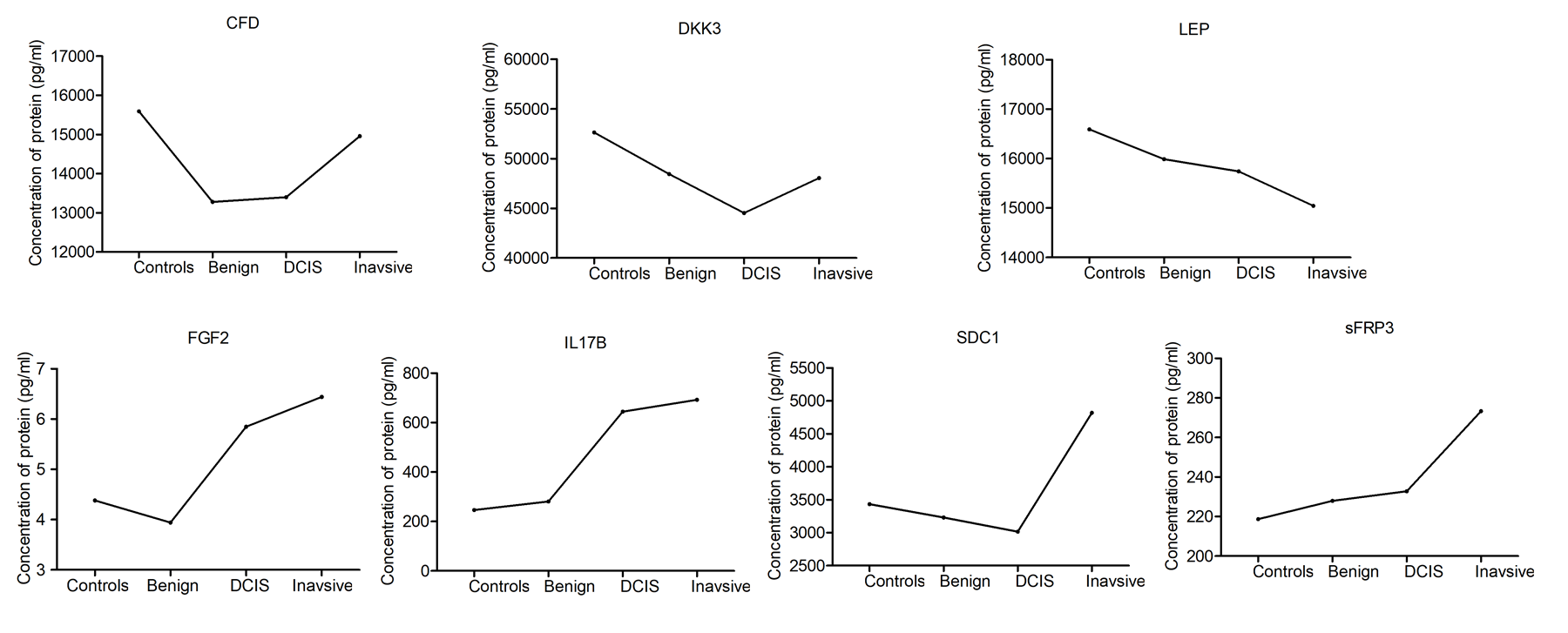
**

**Supplementary Fig S3:** **The median concentration of circulating cell free DNA in controls, benign, DCIS, early, locally advanced and metastatic breast cancer groups. Kruskal Wallis test was used to determine the statistical significance. P<0.05 was considered statistically significant**

**
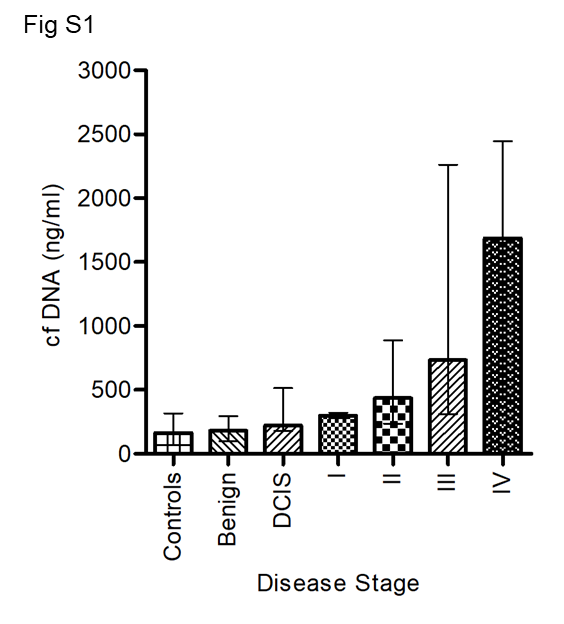
**

**Supplementary Figure S4**

**a MSP analysis of *dact2* in plasma of invasive cases denoted as ‘C’**

**
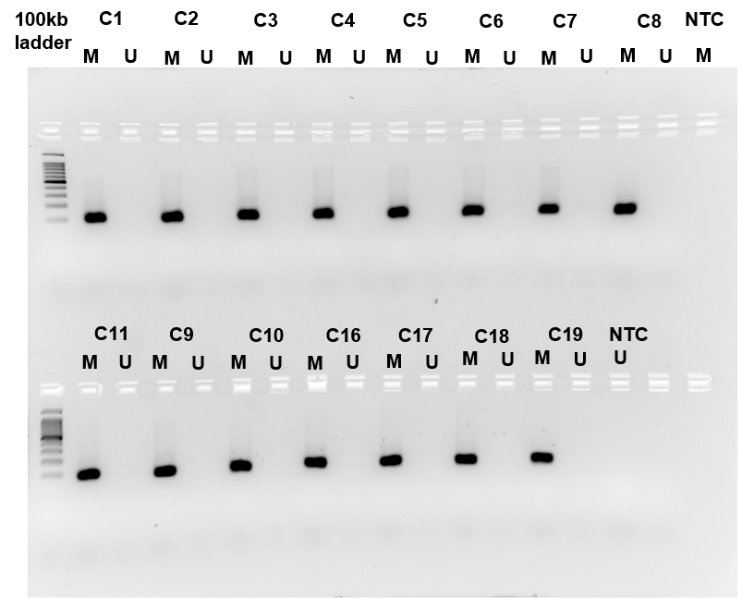
**

**b MSP analysis of *dact2* in plasma of DCIS cases denoted as ‘D’**

**
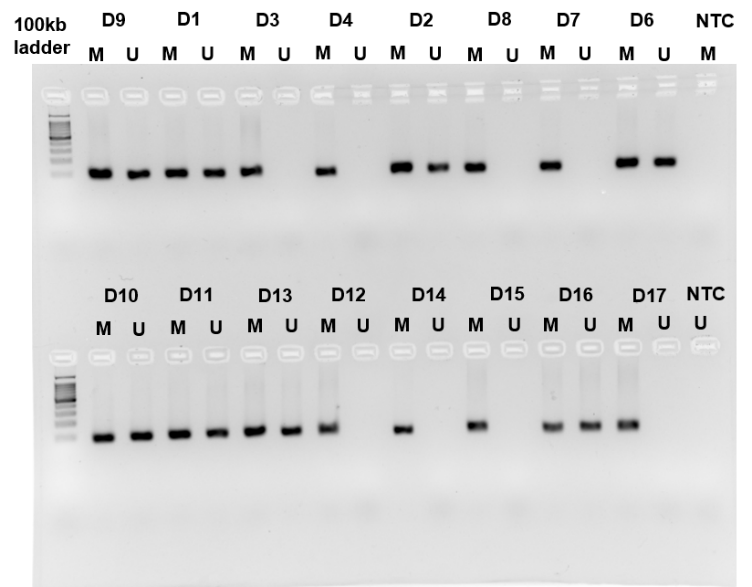
**

**c MSP analysis of *dact2* in plasma of benign breast abnormalities denoted as ‘B’**

**
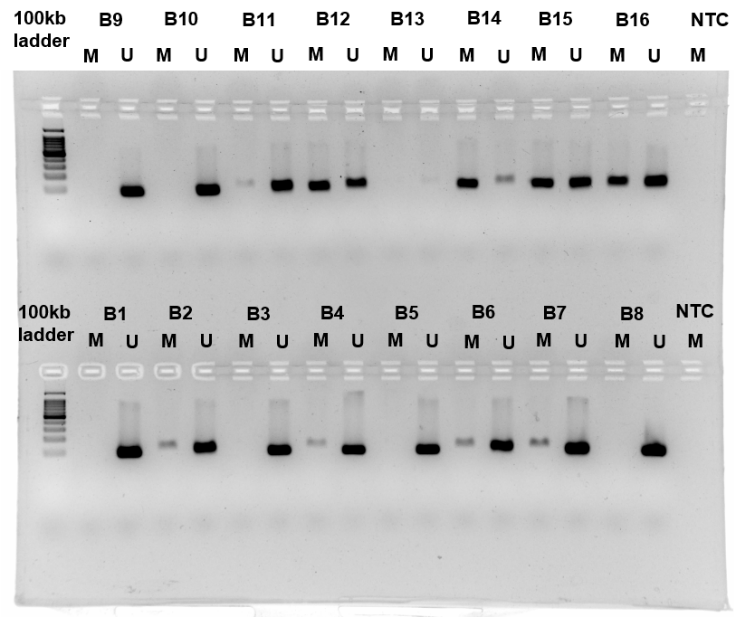
**

**d MSP analysis of *dact2* in plasma of healthy volunteers denoted as ‘O’**

**
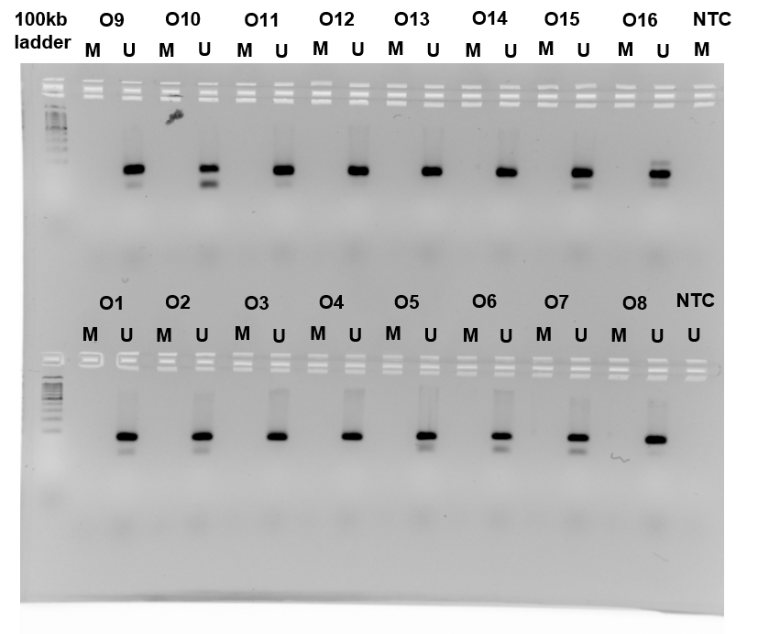
**

**e MSP analysis of *sostdc1* in plasma of invasive cases denoted as ‘C’**

**
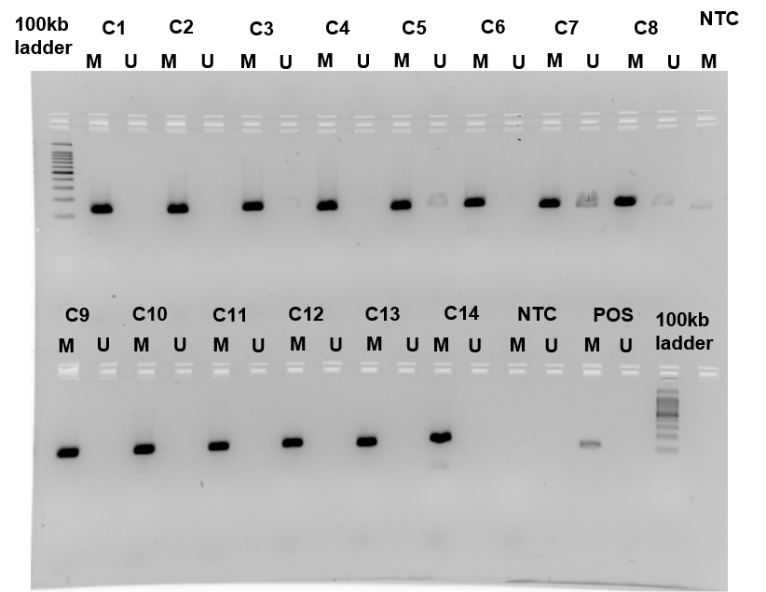
**

**f MSP analysis of *wif1* and *sostdc1* in plasma of DCIS cases denoted as ‘D’**

**
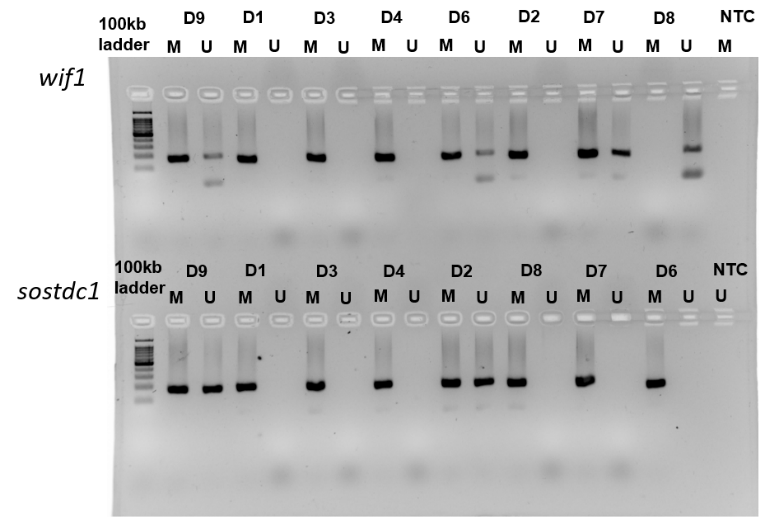
**

**g MSP analysis of *sostdc1* in plasma of benign breast abnormalities denoted as ‘B’**

**
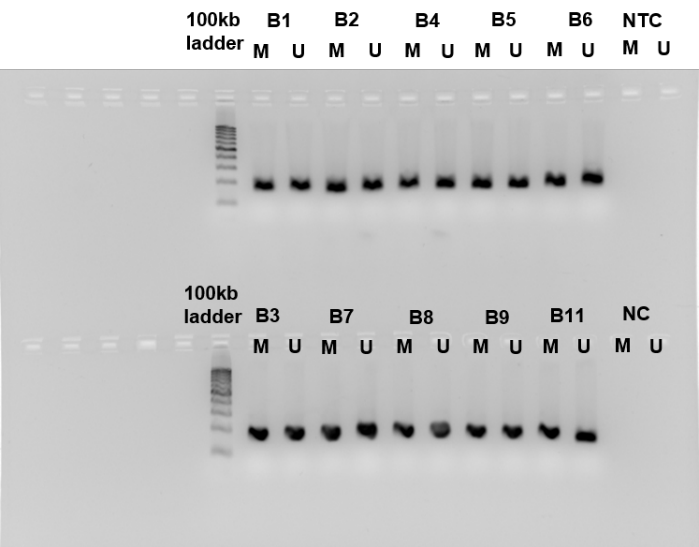
**

**h MSP analysis of *sostdc1* in plasma of healthy volunteers denoted as ‘O’**

**
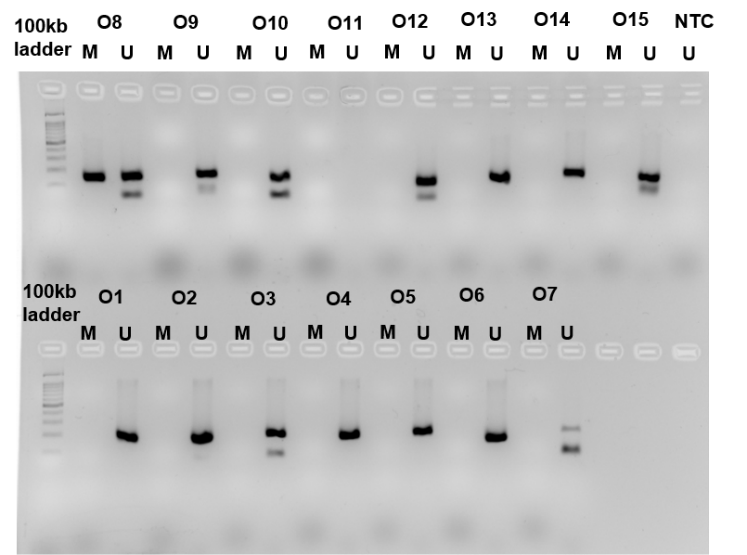
**

**i MSP analysis of *wif1* in plasma of invasive cases denoted as ‘C’**

**
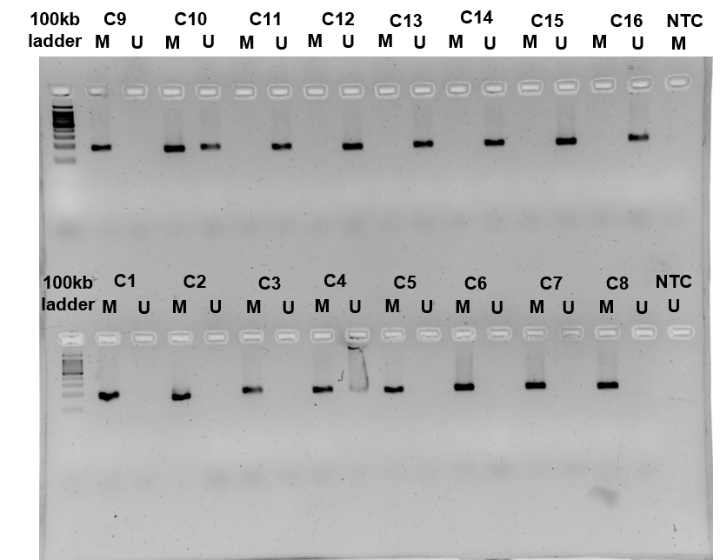
**

**j MSP analysis of *wif1* in plasma of benign breast abnormalities denoted as ‘B’**

**
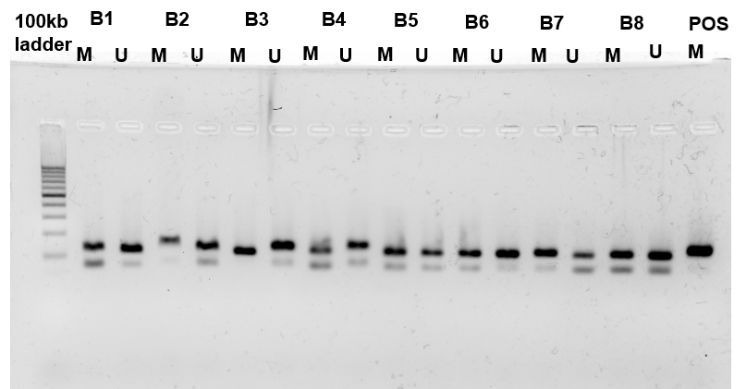
**

**k MSP analysis of *wif1* in plasma of healthy volunteers denoted as ‘O’**

**
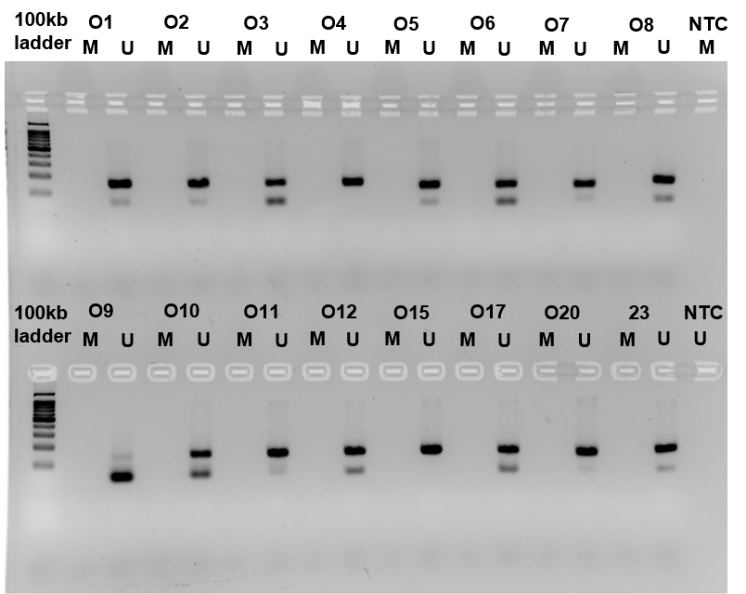
**

**Supplementary Figure S5 Univariate and Multivariate Receiver Operating Characteristic (ROC) Curve Analysis. 4a) Individual ROC curve analysis of SOSTDC1, DACT2, WIF1 in training set (N=317) and test set (N=141), 4b) Combined ROC curve analysis of the three methylation markers in training (N=317) and test sets (N=141). Blue=training set, Red=test set, Black=line of identity (cut-off 0.5), AUC=Area Under the Curve. The combined ROC curve was plotted using the predicted probabilities obtained from binomial logistic regression analysis of the predicted diagnostic model.**

**
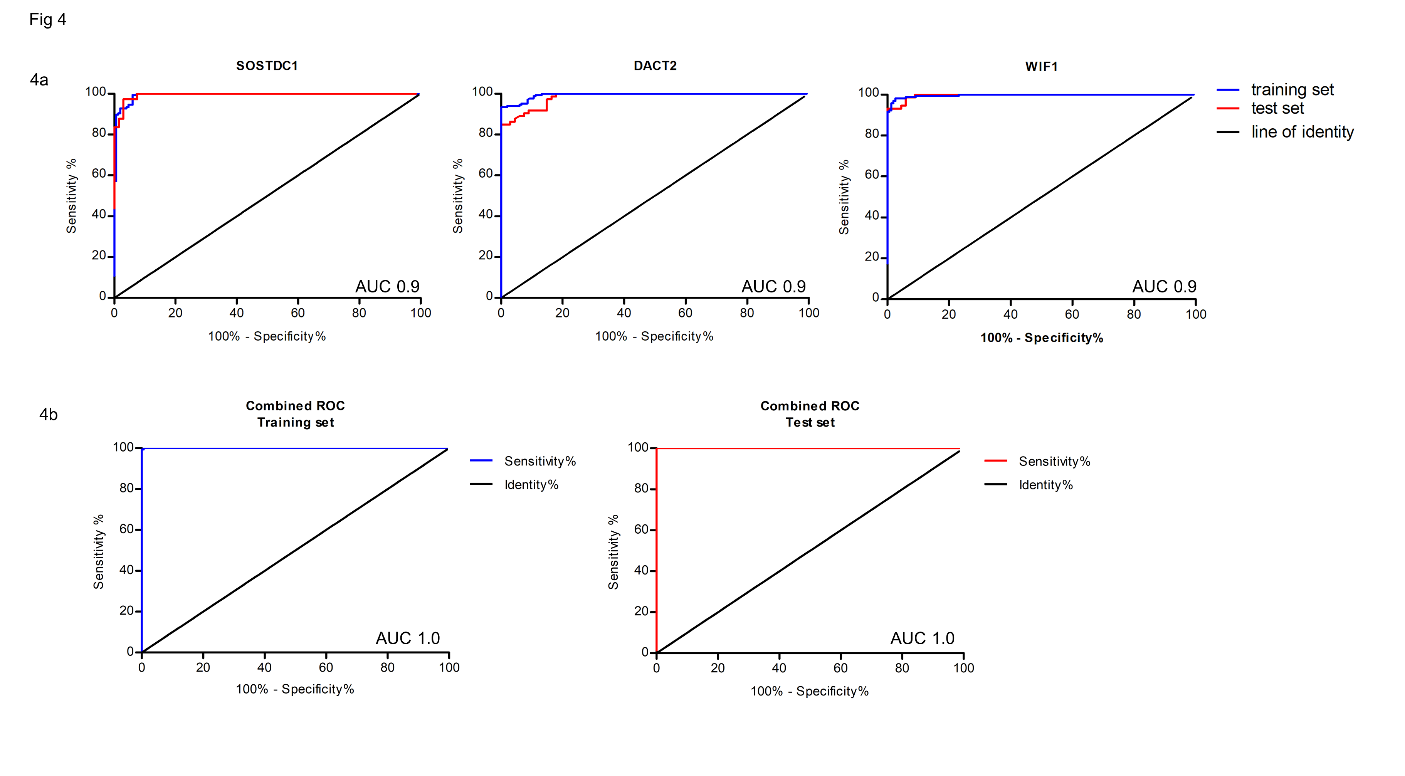
**

**References**

1. Veeck J, Wild PJ, Fuchs T, Schüffler PJ, Hartmann A, Knüchel R, et al. Prognostic relevance of Wnt-inhibitory factor-1 (WIF1) and Dickkopf-3 (DKK3) promoter methylation in human breast cancer. BMC Cancer. 2009;9(Ci):1–13.

2. Li LC, Dahiya R. MethPrimer: Designing primers for methylation PCRs. Bioinformatics. 2002;

3. Rawat A, Gopisetty G, Thangarajan R. E4BP4 is a repressor of epigenetically regulated SOSTDC1 expression in breast cancer cells. Cell Oncol. 2014;

4. Untergasser A, Cutcutache I, Koressaar T, Ye J, Faircloth BC, Remm M, et al. Primer3-new capabilities and interfaces. Nucleic Acids Res. 2012;

5. Li J, Zhang M, He T, Li H, Cao T, Zheng L. Methylation of DACT2 promotes breast cancer development by activating Wnt signaling. 2017;1–10.

6. Ai L, Tao Q, Zhong S, Fields CR, Kim W, Lee MW, et al. Inactivation of Wnt inhibitory factor-1 ( WIF1 ) expression by epigenetic silencing is a common event in breast cancer. 2006;27(7):1341–8.
